# Global, Regional, and National Incidence and Disability-Adjusted Life-Years for Urolithiasis in 195 Countries and Territories, 1990–2019: Results from the Global Burden of Disease Study 2019

**DOI:** 10.3390/jcm12031048

**Published:** 2023-01-29

**Authors:** Juan Li, Yue Zhao, Zhuang Xiong, Guoqiang Yang

**Affiliations:** Department of Urology, The Third Medical Centre, Chinese PLA (People’ s Liberation Army) General Hospital, 69 Yongding Road, Haidian District, Beijing 100853, China

**Keywords:** disability-adjusted life years, global burden, incidence, urolithiasis, prevention

## Abstract

**Purpose:** Urolithiasis is highly prevalent worldwide. The aim of this study was to report the results of the Global Burden of Disease 2019 study on urolithiasis burden estimates grouped by gender, regions, countries or territories, and sociodemographic index (SDI) from 1990 to 2019 globally. **Methods:** We reported detailed estimates and temporal trends of the burden estimates of urolithiasis from 1990 to 2019 in 195 countries and territories and further evaluated the relationship between these estimates and SDI, a composite indicator of income per person, years of education, and fertility as a measurement of country/region socio-economic level. Urolithiasis incidence and disability-adjusted life years by gender, regions, countries or territories, and SDI were reported. The percentage change and estimated annual percentage change of these burden estimates were calculated to quantify temporal trends. **Results:** From 1990 to 2019, the age-standardized incidence rate (ASIR) and disability-adjusted life years (DALYs) of urolithiasis decreased globally by 0.459% and 1.898% per year, respectively. Such a trend of ASIR was prominently due to the decline in the middle, high-middle, and high SDI countries, including Eastern Asia, high-income Eastern Europe, and high-income North America. During this period, these estimates increased in low and low-middle SDI countries, particularly in South Asia, Andean Latin America, and Western Europe. A decline in DALYs was observed in all SDI countries. An approximate positive linear association existed between the burden estimate’s decreased APC and SDI level, except at the high SDI level. Both males and females showed the same trend. **Conclusions:** This study provides comprehensive knowledge of the burden estimate of urolithiasis. Although the burden estimates of urolithiasis showed a global decrease during the past 29 years, this progress has yet to be universal; the increasing trends were observed in countries with low and low-middle SDI countries. Research in these countries is needed and helps with the appropriate allocation of health resources for prevention, screening, and treatment strategies.

## 1. Introduction

Urolithiasis is a highly prevalent disease worldwide with prevalence rates ranging from 1% to 20% [1], it is characterized by significant morbidity, economic costs and days lost from work [2]. In addition, about half of the stone formers have one lifetime recurrence [3]. High recurrence is observed in more than 10% of urolithiasis patients [4]. There is significant variation in rates based on geography, climate, diet style, ethnicity, gender, and age [5]. In countries such as Sweden, Canada or the United States, the prevalence of stones are notably high (more than 10%) [3,6,7]. However, in Asian countries, there was little epidemiologic data about urolithiasis. Yasui et al. [8] reported that the incidence of urolithiasis in Japan was 134.0 per 100,000 person/year, which was significantly lower than that reported in Western countries. Due to the high rates of new and recurrent stones, management of stones is expensive.

Understanding the epidemiology of urolithiasis measures among different regions/nations, and changing trends is crucial for treatment outcome. Furthermore, reliable and accurate statistics on patterns and disease trends in various geographic locations give policy makers the proof they need to allocate resources properly. However, few evaluations have been carried out at the national level, and there is no study addressing the trend in disease burden of urolithiasis. The majority of epidemiological studies of urolithiasis were based on general practice surveys, selected population surveys, or hospitals [9]. For understanding of a nation’s actual demographic status, they are insufficient. A historical evaluation and a comparison of different countries are also impossible. The global burden of disease (GBD) study evaluated the prevalence of urolithiasis in 195 nations and territories, offering a rare chance to comprehend the epidemiology of this condition. By combining several forms of data, it gives a thorough assessment of changes in disease health status. A cycle of continuous quality improvement of this database has additionally led to substantial changes, including new data sources, identification of novel causes of death, and updated methods, which serve as global information freely available for policy makers and public groups seeking to improve human health [10].

Although there were several studies addressing this issue based on GBD 2019 [11,12,13], they did not demonstrate the global disease burden of urolithiasis stratified by sex, countries and territories. Therefore, we have reported an in-depth examination of the global burden of urolithiasis from a complete time series of incidence from 1990 to 2019, with disability-adjusted life years (DALYs), and investigated the disease burden to determine the temporal trends of these estimates at global, regional, and national levels. The relationships of estimates of the global burden of urolithiasis with socioeconomic development level, and measured SDI, were further assessed.

## 2. Methods

### 2.1. Data Sources

The Global Burden of Disease (GBD) is a systematic, scientific effort to quantify the comparative magnitude of health loss due to diseases, injuries, and risk factors by age, sex, and geography over time. The conceptual and analytical framework for GBD 2019, with details of the hierarchy of causes and risk factors, data inputs and processing, and analytical methods, has been published elsewhere [14,15,16]. GBD Results Tool provides the details of different risk factors, causes and impairments related to health in terms of deaths, Disability-Adjusted Life Years (DALYs), Years Lived with Disability (YLDs), Years of Life Lost (YLLs) and prevalence via age, year, gender, and location. Results from the GBD 2019 study, which evaluated 354 causes and 3484 sequelae, were obtained from 195 different nations [14]. These results were generated using a total of 68,781 data sources, including a thorough literature review, hospital and clinical data, surveillance and survey data from various sources, and inpatient and outpatient medical records [14,17]. In our study, data on urolithiasis incidence and DALYs and their uncertainty intervals were curated from GBD 2019 data sources (http://ghdx.healthdata.org/gbd-results-tool (accessed on 1 December 2021)) provided by the Institute for Health Metrics and Evaluation. The present study complies with the Guidelines for Accurate and Transparent Health Estimates Reporting (GATHER) recommendations (Table S1).

### 2.2. Modeling

The major data inputs for the distribution of urolithiasis globally included national health surveys, population representative surveys and cohort studies, and a variety of published and unpublished studies which were described in the appendix (Supplementary Materials, Supplementary Tables S2–S6). In the GBD 2019, urolithiasis was defined as stone formation located anywhere along the genitourinary tract. Using DisMod-MR, version 2.1, a disease modeling computational tool that is the standard GBD modeling approach for non-fatal health outcomes, the incidence of urolithiasis was estimated by region, age, sex, and year (Figure S1). The identification of all accessible data sources, their evaluation for data extraction based on inclusion criteria, the determination of sequelae severity distributions, the incorporation of disability weights to quantify severity, and comorbidity adjustment of sequelae were all steps in the process of estimating the incidence of urolithiasis.

DALYs, a summary measure of total health loss, were calculated for urolithiasis by summing YLLs and YLDs for each location, age, sex, and year (Figure S1). YLD captures years lived with less-than-ideal health because of urolithiasis and was estimated by a multiplication of prevalent cases of urolithiasis and a disability weight [16,18]. YLL is a measure of the years lost owing to premature mortality due to urolithiasis and was based on the remaining life expectancy compared with a reference standard life table at age of death [16].

### 2.3. The Socio-Demographic Index

The Socio-demographic Index (SDI) is a summary measure that estimates a location’s position on a spectrum of development [19]. Briefly, the SDI was computed on the basis of the geometric mean of three indicators: lag-distributed income per capita, educational attainment over the age of 15 years, and total fertility rate in women aged 15–49 years. SDI scores were scaled from 0 to 1, and each location was assigned an SDI score for each year. A total of five SDI quintiles, high, high-middle, middle, low-middle, and low, were selected based on SDI values (Table S7). Average relationships between SDI and incidence and DALYs of urolithiasis were estimated using spline regressions, which were then used to estimate expected values at each level of SDI. The results presented for SDI quintiles in this study reflect each country’s position based on its SDI values in 2019.

### 2.4. Statistical Analysis

To measure the trend in the global burden of urolithiasis, we utilized the age-standardized incidence rate (ASIR), DALYs, percentage change (PC), and estimated annual percentage change (APC) [20]. When comparing populations with varied age structures or for the same population across time when the age profiles change proportionally, standardization is required. By adding the products of the age-specific rates and the number of people in the same age subgroup of the chosen reference standard population, and then dividing the total by the standard population weights, the age-standardized rates according to the direct approach were determined. The GBD world population standard was used for calculation of age-standardized rates. APC is a widely used measure of the ASR trend over a specific time interval. A regression line was fitted to the natural logarithm of the rates. APC and 95% confidential interval (CI) values can also be obtained from the linear regression model [21]. We employed a generalized additive model with Loess smoother on SDI to estimate the associations between SDI and ASIR and DALYs using GBD estimates from all national locations across the years from 1990 to 2019 [22]. Uncertainty intervals (UI) were defined as the 2.5th and 97.5th values of the ordered draws. All statistical analyses were performed using SPSS (Version 23, SPSS Inc., Chicago, IL, USA) and the R program (Version 3.4.4, R core team, Vienna, Austria), with *p*-Values <.001 considered significant.

## 3. Results

### 3.1. Age Standardized Incidence Rate (ASIR) of Urolithiasis

Globally, ASIR of urolithiasis changed from 1146.048 per 100,000 individuals in 1990 to 1031.497 per 100,000 individuals in 2019, representing a shift of −0.459% per year (95%CI: −0.506%–−0.411%) and −9.995% in total. Both male and female showed a decrease in ASIR of urolithiasis, which were −0.557% and −0.312% per year, respectively (Table 1).

**Figure 1:**
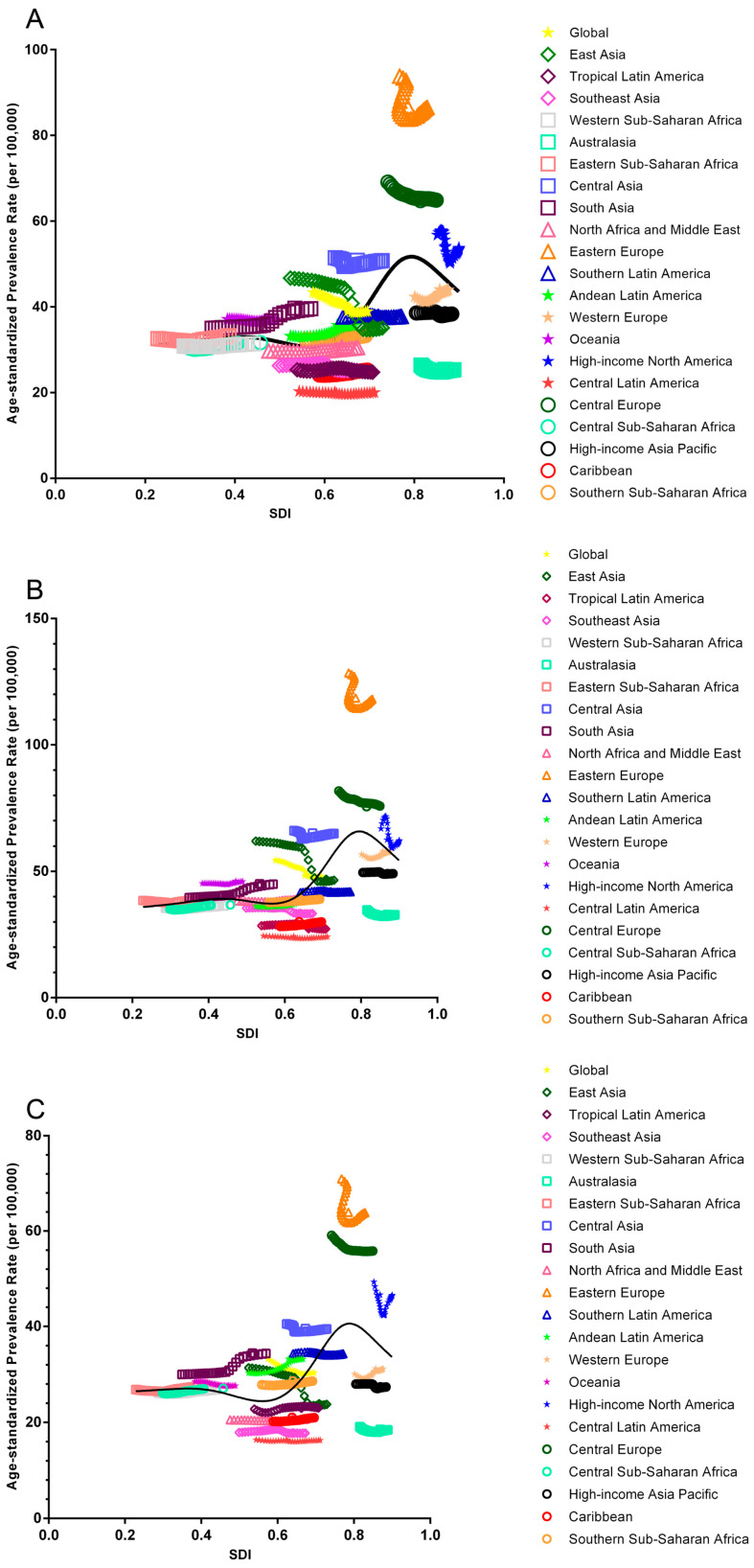
Annual percentage change of age standardized incidence rate of urolithiasis stratified by gender and 21 regions. (**A**) APC of ASIR stratified by SDI levels of both gender; (**B**) APC of ASIR stratified by SDI levels of male; (**C**) APC of ASIR stratified by SDI levels of female.

There was a decreasing trend observed in 12 of 21 regions. The largest decrease in APC was observed in Eastern Asia (−1.396%), followed by high-income Eastern Europe (−0.317%) and high-income North America (−0.305%), which collectively contributed to 73.130% of the decreasing trend. Conversely, an increasing trend, was observed in another 9 regions. The largest increase in APC was detected in South Asia (0.568%), followed by Andean Latin America (0.381%) and Western Europe (0.261%). These three regions contributed 58.815% of the overall increasing trend. (Figure 1 and Table 2).

**Figure 2:**
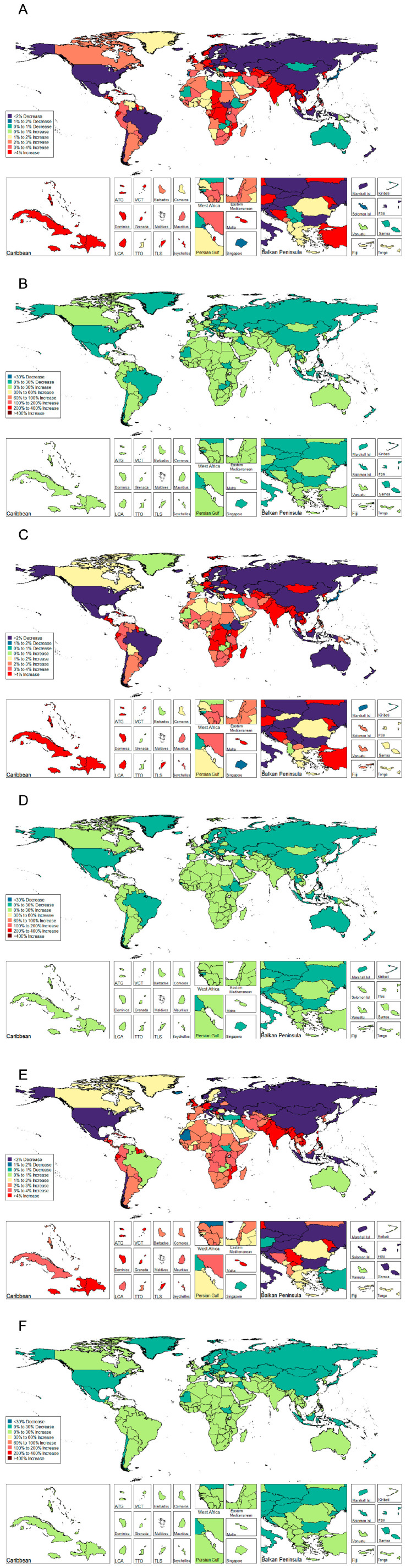
Percentage change and annual percentage change of age standardized incidence rate of urolithiasis stratified by gender and 195 Countries and territories. (**A**) PC of ASIR stratified 195 Countries and territories of both gender; (**B**) APC of ASIR stratified 195 Countries and territories of both gender; (**C**) PC of ASIR stratified 195 Countries and territories of male. (**D**) APC of ASIR stratified 195 Countries and territories of male; (**E**) PC of ASIR stratified 195 Countries and territories of female; (**F**) APC of ASIR stratified 195 Countries and territories of female.

Between 1990 and 2019, APC of ASIR decreased in 53 of the 195 countries, among which statistical significance was reached in 38 countries (71.70%). The top three were China (−1.492%), Indonesia (−0.900%), and New Zealand (−0.673%). Almost three-fourths of the countries or territories (142/195) displayed an increasing trend during the observational period, the majority with statistical significance (89.29%). Territories of Taiwan (a part of China) showed the most pronounced increase with an average of 1.208% per year, followed by Ecuador (APC = 1.006%) and Belgium (APC = 0.891%) (Figure 2 and Table 3).

When stratified by SDI quintiles, ASIR of urolithiasis increased in countries/regions at low and low-middle SDI quintiles but decreased in those at middle, high-middle and high SDI quintiles. There was an approximate positive linear association that existed between the decrease in APC and SDI except at high SDI levels. Both male and female demonstrated the same results. High-middle SDI (APC, −1.165%) and low SDI quintiles (APC, 0.335%) contributed most significantly to the decreasing and increasing trends, respectively (Table 4).

### 3.2. Disability Adjusted Life Years (DALYs) of Urolithiasis

Globally, age-standardized DALYs of urolithiasis decreased by 35.862% from 9.427 per 100,000 individuals in 1990 to 6.046 per 100,000 individuals in 2019, with −1.898% per year (95%CI: −2.117–1.679%). Both male and female showed a decrease in age-standardized DALYs of urolithiasis, which were −1.812% and −2.078% per year, respectively (Table 1).

**Figure 3:**
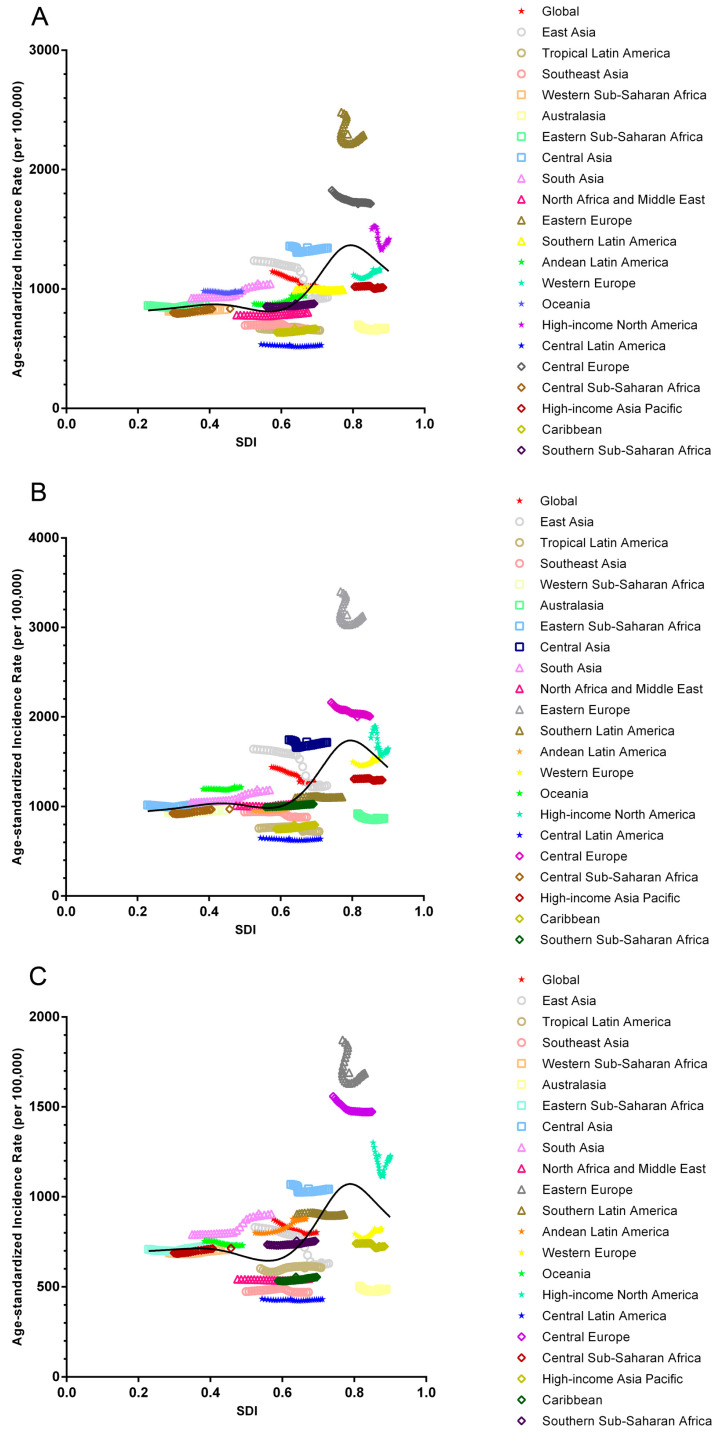
Annual percentage change of disability adjusted life years of urolithiasis stratified by gender and 21 regions. (**A**) APC of DALYs stratified by SDI levels of both gender; (**B**) APC of DALYs stratified by SDI levels of male. (**C**) APC of DALYs stratified by SDI levels of female.

There was a decreasing trend observed in 12 of 21 regions. The largest decrease in APC was observed in Eastern Asia (−4.678%), followed by Central Europe (−2.776%) and Eastern Europe (−1.768%), which collectively contributed to 53.12% of the decreasing trend. Conversely, an increasing trend, was observed in another 9 regions. The largest increase in APC was detected in Tropical Latin America (3.248%), followed by the Caribbean (1.133%) and high-income Asia Pacific (0.670%). These three regions contributed 61.525% of the overall increasing trend (Figure 3 and Table 5).

**Figure 4:**
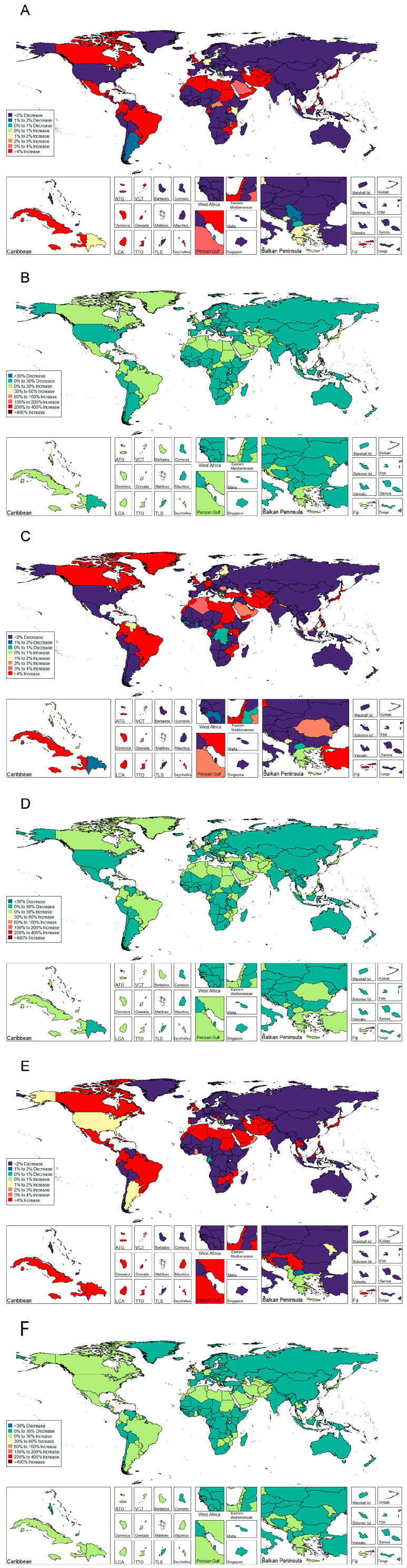
Percentage change and annual percentage change of disability adjusted life years of urolithiasis stratified by gender and 195 Countries and territories. (**A**) PC of DALYs stratified 195 Countries and territories of both gender; (**B**) APC of DALYs stratified 195 Countries and territories of both gender; (**C**) PC of DALYs stratified 195 Countries and territories of male; (**D**) APC of DALYs stratified 195 Countries and territories of male; (**E**) PC of DALYs stratified 195 Countries and territories of female; (**F**) APC of DALYs stratified 195 Countries and territories of female.

Between 1990 and 2019, APC of DALYs decreased in 122 of the 195 countries, among which statistical significance was reached in 101 countries (82.79%). The top three were Bulgaria (−6.073%), American Samoa (−4.974%), and China (−4.811%). Almost one-third of the countries or territories (73/195) displayed an increasing trend during the observational period, the majority with statistical significance (72.60%). Brazil showed the most pronounced increase with an average of 3.279% per year, followed by Trinidad and Tobago (APC = 3.217%) and Armenia (APC = 0.1.995%) (Figure 4 and Table 6).

The associations between global burden estimates of urolithiasis and SDI levels for each of the 21 GBD regions for all individual years between 1990 and 2019 are illustrated in Figure 3 and Table 7. In general, a decreasing trend was observed at all SDI levels, and there was an approximate positive linear association that existed between the decrease in APC and SDI except at the high SDI levels. High-middle SDI (APC, −3.096%) contributed most significantly to the decreasing trends. Both male and female demonstrated a similar demographic pattern.

## 4. Discussion

Based on the GBD 2019 data, we comprehensively assessed the recent burden estimates as well as temporal trends in urolithiasis from 1990 to 2019 at the global, regional, and national levels. During the study period, the global urolithiasis burden decreased as measured by ASIR and DALYs. However, the temporal trends of these burden estimates varied considerably by SDI levels and regions. The ASIR decrease in urolithiasis was observed in the middle, high middle, and high SDI countries, but an increase was shown in low and low middle SDI countries. A decline in DALYs was observed in all SDI levels. Additionally, an approximate positive linear association existed between the decreased APC of burden estimates and SDI, except for at the high SDI levels.

This study showed a slight decline in the incidence of urolithiasis globally for both genders, consistent with several previous evaluations of regional trends in urolithiasis. A recent population-based study from Rochester showed the incidence rates might have decreased in males and reached a plateau in females since 1990 [23]. This study reported relatively stable incidence rates from 1970 to 2000 and a downward trend in the overall incidence of kidney stones in the Caucasian population [23]. Numerous previous data reported that urolithiasis prevalence in most countries has been rising in recent decades [3,7,8,24,25], such as the United States, New Zealand, Germany, and Japan. While the incidence trend was slightly decreased or stable, it implies that new urolithiasis cases increased more slowly.

Although the consequences are not life-threatening in most stone patients, it is a significant cause of morbidity, hospitalization, and days lost from work [26]. There has been a significant decrease in the DALYs of urolithiasis globally, and it decreased linearly with SDI except for high SDI countries. From 1990 to 2019, Global DALYs of urolithiasis, with 122 of 195 countries or territories, had improved. Over the last three decades, this decreasing pattern in the age-standardized DALY rate of urolithiasis may be partly attributable to surgical innovations and better treatment guidelines [27]. These advances have made interventions safe, effective, and associated with shorter recovery duration and lesser discomfort [28].

There is significant geographic variation in urolithiasis incidence worldwide. Even though throughout a country, the incidence may have a drastic range [5]. The variation in demography is impacted by many factors, such as climate, ethnicity, environmental factors, availability of medical practice, dietary styles, and age distribution; these factors interact in complex ways. This study observed a decreasing trend in 12 of 21 regions. The most significant decrease in APC was observed in Eastern Asia, followed by high-income Eastern Europe and high-income North America, collectively contributing to 73.130% of the decreasing trend. In addition, the APC of ASIR decreased in 53 of the 195 countries; the top three were China, Indonesia, and New Zealand. This decreasing trend has been influenced by some regions, particularly in populous East Asia. For example, as the most populous country in the world, China has experienced a remarkable decline. In the last decades, the diet structure of China has greatly changed, and the consumption of fruits and vegetables is on the rise, which are protective factors for urolithiasis development [29]. This could partially help to explain the decreasing trend.

However, an increasing trend was observed in the other nine regions. The most significant increase in APC was detected in South Asia, followed by Andean Latin America and Western Europe. These three regions contributed 58.815% of the overall increasing trend. In addition, between 1990 and 2019, almost three-fourths of the countries or territories displayed a rising trend during the observational period, the majority with statistical significance. The territories of Taiwan (a part of China) showed the most pronounced increase, followed by Ecuador and Belgium. The progress in diagnostic procedures, such as sonography, has led to a significant improvement in early diagnosis of asymptomatic urolithiasis, which may increase the trend in low and low-middle SDI nations [30]. Significant changes in nutritional and environmental factors might also lead to progress in the burden of urolithiasis [30].

While most countries in the low and low-middle SDI quintiles showed an increase in ASIR, these values declined in the middle, high-middle, and high SDI quintile countries. Between-country variations in factors, such as socioeconomic status (per-capita income, fertility, and education levels), access to prevention, diagnosis, and treatment facilities, and differences in clinical practice, could further lead to heterogeneity in these burden estimates. Socioeconomic status (SES) differences in health outcomes are among the most consistent epidemiological findings [31]. An earlier ecological study also reported an association of diversity of income and education levels with incidence and mortality differences of disease in each region [32], patients with higher SES levels might have less unhealthy living behavior than lower SES patients [32]. Furthermore, compared to countries with low income, high-income countries have more advocacy, media attention, and funding for the prevention and treatment of disease [33]. Therefore, to further reduce the disease burden, more regions, especially countries with low or middle SDI, should consider increasing the investment in health careers [34]. Changes in socioeconomic conditions over time, and the subsequent changes in dietary styles, have affected not only the incidence rate but also the location and composition of stones [2]. In addition, the observed variation in urolithiasis estimated burden among the SDI quintiles levels was not only due to differences in socioeconomic status but also to differences in genetic background, lifestyles, and exposure to environmental and nutritional factors.

In addition, due to global warming from climate change, it is expected that the prevalence of kidney stone disease will increase due to more significant insensible water losses, resulting in more concentrated urine and altered urinary flow. In line with this, Kaufman et al. found that an increased burden of kidney stone disease on healthcare systems attributed to climate warming is very likely [35]. Especially the burden of greenhouse gas emissions was more prominently observed in low-income countries [36], which may be another plausible reason for explaining the disease burden trend discrepancy between various income levels of regions and countries.

The Asian–Africa stone-forming belt includes the Philippines, Indonesia, Thailand, Myanmar, India, Pakistan, Iran, the United Arab Emirates, Saudi Arabia, Egypt, and Sudan. In this area, urolithiasis was detected in all age groups, with prevalence ranging from 4% to 20% [37]. The higher prevalence in these stone-forming belt countries is possibly determined by the high consanguinity among ethnic groups [38]. In the current study, we found that this stone belt still exists. However, the estimated burden of a few countries declined, and the decreased trend was significant in Indonesia, Thailand, and Sudan.

There were several limitations of the study. First, GBD estimates are a combination of data and largely depend on the quality and quantity of data used in the modeling [10]. The health surveys and other data systems in different countries result in wide uncertainty in these estimates. Several statistical procedures have been developed to address this flaw, including modeling based on regional patterns and disease-specific covariates [39]. Furthermore, differences in data collection practices and coding systems and the quality of data sources remain major challenges. However, the GBD 2019 study has made a substantial effort to solve these difficulties in the methodological framework, including applying corrections for under-registration and garbage code redistribution algorithms [40]. Secondly, given the misclassification of urolithiasis and the adoption of different disease coding systems in the input data sources, we failed to estimate temporal trends in the burden of urolithiasis stratified by stone location and composition. Thirdly, SDI utility is restricted in countries with income inequality. The applicability of SDI could therefore be enhanced by taking into account social heterogeneity within countries [41]. However, data from GBD is the most thorough and standardized when compared to other sources because it provides complete time series and outcomes at the country level. This is useful for policymakers who need to effectively distribute the limited resources in their healthcare systems.

## 5. Conclusions

Since urolithiasis is a common disease worldwide, elucidating the trends and burden estimates over time is essential to establish policies and accurately set priorities for action. The GBD 2019 study provides an opportunity to assess the latest evidence, and monitor these trends to determine where interventions exert an effect. Our findings collectively indicate that while progress has been made in reducing the global burden of urolithiasis in the middle, high-middle, and high-SDI countries, more effective prevention strategies are required for low and low-middle SDI countries.

## Figures and Tables

**Table 1:** The global age standardized incidence rate and disability adjusted life years of urolithiasis from 1990 to 2019 stratified by gender.

	ASIR			DALYs		
Year	Both Male and Female	Male	Female	Both Male and Female	Male	Female
**1990**	1146.047641	1440.793066	875.8114857	9.427001255	11.18863773	7.973054209
**1991**	1140.911218	1436.61929	868.5097849	9.39061477	11.16270689	7.922430885
**1992**	1135.762233	1431.9645	861.7192101	9.36658052	11.09585561	7.930221913
**1993**	1130.191462	1426.5021	855.0269892	9.299745192	10.99529361	7.881904971
**1994**	1124.645275	1420.736097	848.901434	9.053485315	10.73258633	7.638401684
**1995**	1119.436554	1415.011279	843.6554232	8.692092247	10.34541661	7.285534651
**1996**	1113.859253	1408.393382	838.7490017	8.282838434	9.869189374	6.923735513
**1997**	1107.69147	1400.543025	833.931449	8.005927994	9.625529321	6.606542967
**1998**	1101.155628	1392.024039	829.1017686	7.757849186	9.323976626	6.39918849
**1999**	1095.041447	1383.898419	824.7663553	7.603703949	9.1440201	6.264852915
**2000**	1090.020906	1377.162259	821.2682917	7.442599032	9.012731993	6.06963117
**2001**	1086.013963	1371.772109	818.4771954	7.251061201	8.859252356	5.842025802
**2002**	1082.627938	1367.045562	816.2512963	7.081799647	8.698538836	5.660230101
**2003**	1079.422137	1362.43179	814.2720769	6.912126255	8.540729982	5.476002512
**2004**	1076.408171	1357.917612	812.5774435	6.759182321	8.378038779	5.326008996
**2005**	1073.399559	1353.298749	811.0104047	6.640680704	8.23488217	5.222382904
**2006**	1065.985207	1341.19763	808.1331063	6.449663697	8.005666955	5.061498996
**2007**	1052.913051	1319.364923	803.6658621	6.303375455	7.810608222	4.956258738
**2008**	1038.240731	1294.641594	798.9102725	6.233267481	7.704416674	4.917888338
**2009**	1026.320337	1274.275091	795.3064952	6.124649977	7.531218857	4.867998357
**2010**	1021.561136	1265.710553	794.3282972	6.079312489	7.433786439	4.868709668
**2011**	1021.931662	1265.637737	795.1469297	6.009475453	7.325908862	4.830072279
**2012**	1022.798159	1266.314351	796.2151329	5.979276847	7.270361007	4.819140076
**2013**	1024.003667	1267.443282	797.5020103	5.98219769	7.238329177	4.851921944
**2014**	1025.494809	1268.944095	798.9300114	6.027458681	7.259450318	4.917627711
**2015**	1027.278721	1270.930727	800.47074	6.09657588	7.317248455	4.995490077
**2016**	1029.251288	1273.264415	802.0485335	6.113928135	7.331595153	5.015602337
**2017**	1029.342488	1274.353335	802.1264334	6.124817645	7.330605333	5.014520307
**2018**	1030.366188	1275.154675	803.4326121	6.163431149	7.286751188	4.953148863
**2019**	1031.497187	1276.066795	803.7609031	6.046272099	7.267762028	4.946559974

Abbreviations: Age standardized Incidence rate, ASIR; Disability adjusted life years, DALYs.

**Table 2:** Percentage change and annual percentage change of age standardized incidence rate of urolithiasis stratified by gender and 21 regions.

	Both Male and Female					Male					Female				
	PC	APC	95%CI	95%CI	*p*-Value	PC	APC	95%CI	95%CI	*p*-Value	PC	APC	95%CI	95%CI	*p*-Value
**Central Asia**	−1.065	−0.006	−0.063	0.050	0.818	−1.530	−0.010	−0.080	0.060	0.772	−2.341	−0.076	−0.127	−0.025	0.005
**Eastern Asia**	−24.330	−1.396	−1.653	−1.138	<0.001	−24.363	−1.402	−1.675	−1.130	<0.001	−23.715	−1.335	−1.557	−1.114	<0.001
**High-income Asia Pacific**	−0.485	−0.062	−0.091	−0.033	<0.001	−0.854	−0.066	−0.087	−0.045	<0.001	−2.032	−0.130	−0.171	−0.088	<0.001
**South Asia**	13.301	0.568	0.481	0.656	<0.001	13.904	0.564	0.490	0.638	<0.001	14.777	0.660	0.550	0.770	<0.001
**Southeast Asia**	−4.006	−0.253	−0.329	−0.177	<0.001	−5.788	−0.336	−0.413	−0.259	<0.001	−0.610	−0.099	−0.173	−0.026	0.010
**Central Europe**	−6.216	−0.196	−0.229	−0.162	<0.001	−7.562	−0.237	−0.263	−0.210	<0.001	−5.417	−0.182	−0.226	−0.137	<0.001
**Eastern Europe**	−7.412	−0.317	−0.434	−0.200	<0.001	−7.647	−0.336	−0.454	−0.217	<0.001	−9.720	−0.393	−0.529	−0.257	<0.001
**Western Europe**	4.148	0.262	0.219	0.304	<0.001	2.298	0.188	0.148	0.229	<0.001	3.852	0.263	0.212	0.314	<0.001
**Andean Latin America**	7.773	0.382	0.340	0.424	<0.001	6.184	0.317	0.275	0.358	<0.001	9.655	0.456	0.411	0.501	<0.001
**Central Latin America**	−1.064	−0.029	−0.074	0.017	0.205	−0.919	−0.038	−0.096	0.020	0.193	−0.571	0.011	−0.019	0.041	0.464
**Southern Latin America**	0.496	−0.032	−0.052	−0.013	0.002	0.874	−0.016	−0.035	0.003	0.098	0.102	−0.051	−0.073	−0.028	<0.001
**Tropical Latin America**	−2.436	−0.084	−0.142	−0.025	0.007	−4.542	−0.285	−0.368	−0.202	<0.001	0.489	0.157	0.094	0.221	<0.001
**High-income North America**	−5.087	−0.305	−0.491	−0.120	0.002	−6.431	−0.572	−0.779	−0.365	<0.001	−5.464	0.001	−0.207	0.209	0.992
**Central Sub-Saharan Africa**	4.118	0.194	0.154	0.234	<0.001	4.962	0.225	0.179	0.270	<0.001	3.656	0.169	0.140	0.197	<0.001
**Eastern Sub-Saharan Africa**	2.022	0.094	0.042	0.146	0.001	2.130	0.097	0.039	0.155	0.002	2.891	0.136	0.088	0.184	<0.001
**Southern Sub-Saharan Africa**	2.884	0.127	0.109	0.144	<0.001	3.415	0.135	0.129	0.141	<0.001	2.876	0.135	0.116	0.154	<0.001
**Western Sub-Saharan Africa**	1.653	0.078	0.055	0.101	<0.001	2.655	0.111	0.080	0.142	<0.001	2.451	0.124	0.095	0.154	<0.001
**North Africa and Middle East**	3.135	0.140	0.110	0.169	<0.001	3.475	0.163	0.136	0.191	<0.001	1.180	0.037	0.007	0.067	0.016
**Oceania**	−0.158	−0.013	−0.041	0.015	0.363	1.933	0.070	0.039	0.101	<0.001	−3.025	−0.143	−0.166	−0.120	<0.001
**Australasia**	−3.774	−0.067	−0.136	0.001	0.054	−5.276	−0.127	−0.200	−0.054	0.001	−3.053	−0.035	−0.106	0.036	0.315
**Caribbean**	5.131	0.216	0.197	0.235	<0.001	6.675	0.268	0.248	0.288	<0.001	4.064	0.176	0.158	0.195	<0.001

Abbreviations: PC: percentage change; APC: annual percentage change. *p* < 0.001 considered significant.

**Table 3:** Percentage change and annual percentage change of age standardized incidence rate of urolithiasis stratified by gender and 195 Countries and territories.

		Both Male and Female					Male					Female				
Number	Countries and Territories	PC	APC	95%CI	95%CI	*p*-Value	PC	APC	95%CI	95%CI	*p*-Value	PC	APC	95%CI	95%CI	*p*-Value
1	**Afghanistan**	2.105	0.106	0.078	0.134	<0.001	2.540	0.116	0.092	0.140	<0.001	2.374	0.094	0.075	0.114	<0.001
2	**Albania**	1.031	0.057	0.034	0.080	<0.001	0.925	0.066	0.034	0.098	<0.001	2.053	0.079	0.064	0.093	<0.001
3	**Algeria**	2.295	0.098	0.087	0.108	<0.001	1.942	0.089	0.077	0.102	<0.001	2.043	0.077	0.068	0.086	<0.001
4	**American Samoa**	−10.603	−0.520	−0.573	−0.466	<0.001	−8.667	−0.430	−0.484	−0.376	<0.001	−11.115	−0.548	−0.590	−0.506	<0.001
5	**Andorra**	0.186	−0.037	−0.052	−0.022	<0.001	0.859	0.006	−0.012	0.024	0.491	2.008	0.047	0.035	0.060	<0.001
6	**Angola**	2.561	0.131	0.105	0.158	<0.001	4.465	0.200	0.173	0.227	<0.001	3.386	0.162	0.136	0.188	<0.001
7	**Antigua and Barbuda**	6.458	0.244	0.232	0.256	<0.001	5.941	0.226	0.213	0.238	<0.001	5.040	0.195	0.189	0.202	<0.001
8	**Argentina**	2.269	0.087	0.073	0.101	<0.001	2.539	0.097	0.085	0.109	<0.001	2.059	0.077	0.064	0.090	<0.001
9	**Armenia**	−5.537	−0.208	−0.280	−0.136	<0.001	−4.310	−0.145	−0.218	−0.071	<0.001	−7.671	−0.332	−0.393	−0.272	<0.001
10	**Australia**	−0.339	0.028	−0.009	0.064	0.129	−2.071	−0.035	−0.083	0.012	0.140	0.518	0.059	0.032	0.087	<0.001
11	**Austria**	6.372	0.277	0.012	0.541	0.041	7.061	0.295	−0.080	0.671	0.118	−0.339	−0.002	−0.080	0.076	0.954
12	**Azerbaijan**	5.502	0.280	0.207	0.352	<0.001	4.577	0.254	0.148	0.360	<0.001	3.702	0.200	0.144	0.257	<0.001
13	**Bahrain**	3.254	0.175	0.154	0.196	<0.001	2.001	0.090	0.083	0.096	<0.001	1.285	0.038	0.033	0.042	<0.001
14	**Bangladesh**	8.883	0.327	0.296	0.359	<0.001	11.902	0.436	0.390	0.483	<0.001	6.904	0.267	0.235	0.299	<0.001
15	**Barbados**	2.702	0.140	0.126	0.154	<0.001	0.566	0.083	0.050	0.116	<0.001	2.920	0.134	0.117	0.151	<0.001
16	**Belarus**	5.666	0.227	0.128	0.326	<0.001	7.651	0.299	0.199	0.399	<0.001	2.929	0.145	0.031	0.258	0.014
17	**Belgium**	17.111	0.891	0.628	1.155	<0.001	17.327	0.912	0.626	1.198	<0.001	13.416	0.731	0.515	0.947	<0.001
18	**Belize**	6.754	0.249	0.243	0.254	<0.001	7.918	0.285	0.275	0.294	<0.001	4.740	0.179	0.158	0.200	<0.001
19	**Benin**	2.202	0.097	0.070	0.123	<0.001	1.602	0.060	0.042	0.079	<0.001	2.936	0.139	0.110	0.167	<0.001
20	**Bermuda**	3.960	0.179	0.165	0.193	<0.001	5.262	0.216	0.201	0.231	<0.001	1.910	0.104	0.087	0.121	<0.001
21	**Bhutan**	8.416	0.309	0.287	0.331	<0.001	8.959	0.342	0.311	0.372	<0.001	6.192	0.235	0.216	0.254	<0.001
22	**Bolivia**	2.084	0.098	0.084	0.112	<0.001	1.169	0.069	0.053	0.084	<0.001	2.885	0.128	0.114	0.141	<0.001
23	**Bosnia and Herzegovina**	4.153	0.165	0.154	0.176	<0.001	4.099	0.174	0.161	0.188	<0.001	3.308	0.126	0.119	0.133	<0.001
24	**Botswana**	3.260	0.133	0.107	0.159	<0.001	3.197	0.126	0.104	0.148	<0.001	2.787	0.120	0.100	0.140	<0.001
25	**Brazil**	−2.582	−0.089	−0.149	−0.029	0.005	−4.772	−0.298	−0.383	−0.212	<0.001	0.438	0.159	0.094	0.224	<0.001
26	**Brunei**	−3.843	−0.153	−0.175	−0.131	<0.001	−4.072	−0.174	−0.183	−0.165	<0.001	1.050	0.047	0.038	0.056	<0.001
27	**Bulgaria**	−15.062	−0.616	−0.754	−0.478	<0.001	−16.907	−0.715	−0.859	−0.570	<0.001	−12.332	−0.488	−0.610	−0.366	<0.001
28	**Burkina Faso**	1.022	0.050	0.038	0.062	<0.001	0.922	0.041	0.026	0.055	<0.001	1.711	0.089	0.070	0.109	<0.001
29	**Burundi**	3.680	0.139	0.085	0.192	<0.001	0.447	0.005	−0.058	0.068	0.873	2.964	0.125	0.078	0.171	<0.001
30	**Cambodia**	5.173	0.211	0.165	0.256	<0.001	4.426	0.189	0.135	0.243	<0.001	4.206	0.161	0.132	0.190	<0.001
31	**Cameroon**	1.129	0.060	0.037	0.083	<0.001	1.012	0.045	0.027	0.063	<0.001	1.037	0.066	0.036	0.097	<0.001
32	**Canada**	2.296	0.105	0.097	0.114	<0.001	1.780	0.091	0.082	0.099	<0.001	1.498	0.068	0.057	0.079	<0.001
33	**Cape Verde**	4.380	0.183	0.152	0.214	<0.001	3.366	0.149	0.118	0.180	<0.001	2.315	0.106	0.083	0.129	<0.001
34	**Central African Republic**	4.741	0.199	0.167	0.231	<0.001	5.124	0.204	0.171	0.236	<0.001	3.754	0.166	0.136	0.195	<0.001
35	**Chad**	4.400	0.183	0.156	0.210	<0.001	2.836	0.111	0.085	0.138	<0.001	3.213	0.151	0.119	0.183	<0.001
36	**Chile**	−4.029	−0.326	−0.383	−0.269	<0.001	−3.394	−0.291	−0.347	−0.236	<0.001	−4.931	−0.373	−0.436	−0.310	<0.001
37	**China**	−26.411	−1.492	−1.764	−1.220	<0.001	−26.565	−1.500	−1.787	−1.213	<0.001	−25.503	−1.424	−1.656	−1.191	<0.001
38	**Colombia**	3.242	0.123	0.100	0.146	<0.001	3.749	0.144	0.122	0.166	<0.001	3.511	0.129	0.109	0.150	<0.001
39	**Comoros**	1.315	0.057	0.011	0.103	0.018	1.757	0.071	0.022	0.121	0.006	2.113	0.084	0.046	0.121	<0.001
40	**Congo**	4.186	0.179	0.154	0.204	<0.001	3.259	0.134	0.108	0.160	<0.001	3.137	0.144	0.124	0.165	<0.001
41	**Costa Rica**	1.848	0.073	0.054	0.093	<0.001	2.726	0.106	0.086	0.127	<0.001	2.157	0.085	0.061	0.109	<0.001
42	**Cote d’Ivoire**	1.304	0.053	0.027	0.078	<0.001	1.317	0.034	0.011	0.057	0.005	1.942	0.092	0.069	0.115	<0.001
43	**Croatia**	−11.731	−0.605	−0.711	−0.499	<0.001	−15.729	−0.749	−0.947	−0.551	<0.001	−8.157	−0.480	−0.541	−0.419	<0.001
44	**Cuba**	5.371	0.221	0.192	0.251	<0.001	7.643	0.291	0.263	0.319	<0.001	3.718	0.169	0.141	0.198	<0.001
45	**Cyprus**	−1.128	−0.162	−0.246	−0.077	0.001	1.872	0.027	0.004	0.050	0.021	−7.131	−0.546	−0.765	−0.327	<0.001
46	**Czech Republic**	−9.462	−0.288	−0.381	−0.196	<0.001	−10.369	−0.258	−0.383	−0.132	<0.001	−11.376	−0.457	−0.528	−0.385	<0.001
47	**Democratic Republic of the Congo**	4.480	0.210	0.162	0.257	<0.001	5.193	0.238	0.183	0.294	<0.001	3.770	0.172	0.141	0.203	<0.001
48	**Denmark**	1.823	−0.039	−0.118	0.041	0.325	0.480	−0.123	−0.238	−0.008	0.037	1.210	−0.001	−0.022	0.020	0.901
49	**Djibouti**	0.180	−0.011	−0.029	0.008	0.257	−0.960	−0.050	−0.073	−0.028	<0.001	0.373	0.013	−0.012	0.039	0.287
50	**Dominica**	7.734	0.301	0.288	0.315	<0.001	6.972	0.273	0.249	0.296	<0.001	4.330	0.173	0.157	0.190	<0.001
51	**Dominican Republic**	5.455	0.229	0.208	0.249	<0.001	6.870	0.277	0.250	0.305	<0.001	4.285	0.170	0.157	0.183	<0.001
52	**Ecuador**	20.409	1.006	0.889	1.124	<0.001	15.411	0.829	0.721	0.936	<0.001	26.535	1.209	1.076	1.342	<0.001
53	**Egypt**	2.670	0.103	0.090	0.117	<0.001	2.474	0.100	0.088	0.113	<0.001	1.959	0.072	0.060	0.083	<0.001
54	**El Salvador**	3.046	0.116	0.094	0.138	<0.001	5.040	0.182	0.161	0.204	<0.001	2.796	0.116	0.095	0.138	<0.001
55	**Equatorial Guinea**	7.297	0.321	0.284	0.359	<0.001	8.401	0.346	0.288	0.404	<0.001	5.772	0.262	0.233	0.291	<0.001
56	**Eritrea**	2.160	0.039	0.012	0.066	0.006	1.512	−0.033	−0.071	0.006	0.097	3.915	0.144	0.118	0.169	<0.001
57	**Estonia**	−3.388	−0.165	−0.265	−0.064	0.002	−6.013	−0.303	−0.384	−0.222	<0.001	−4.609	−0.174	−0.295	−0.053	0.006
58	**Ethiopia**	−0.327	0.031	−0.062	0.123	0.501	−2.463	−0.039	−0.156	0.079	0.506	2.128	0.133	0.055	0.210	0.002
59	**Federated States of Micronesia**	−3.597	−0.158	−0.169	−0.146	<0.001	1.147	0.034	0.006	0.062	0.019	−9.703	−0.418	−0.453	−0.384	<0.001
60	**Fiji**	1.621	0.073	0.054	0.092	<0.001	2.101	0.085	0.067	0.103	<0.001	1.702	0.070	0.051	0.089	<0.001
61	**Finland**	−6.480	−0.242	−0.593	0.110	0.169	−8.061	−0.334	−0.655	−0.011	0.043	−8.630	−0.292	−0.683	0.101	0.139
62	**France**	2.136	0.058	0.037	0.079	<0.001	0.697	0.011	−0.018	0.040	0.448	2.543	0.072	0.061	0.082	<0.001
63	**Gabon**	2.436	0.098	0.093	0.103	<0.001	1.942	0.068	0.060	0.075	<0.001	2.201	0.099	0.088	0.109	<0.001
64	**Georgia**	−5.470	−0.295	−0.355	−0.234	<0.001	−7.376	−0.386	−0.457	−0.315	<0.001	−5.282	−0.275	−0.323	−0.228	<0.001
65	**Germany**	17.760	0.833	0.722	0.944	<0.001	16.550	0.805	0.681	0.929	<0.001	13.405	0.667	0.568	0.766	<0.001
66	**Ghana**	1.944	0.072	0.058	0.085	<0.001	2.850	0.094	0.082	0.107	<0.001	3.080	0.142	0.116	0.169	<0.001
67	**Greece**	1.826	0.033	0.018	0.047	<0.001	1.508	0.017	−0.003	0.037	0.100	1.587	0.031	0.021	0.040	<0.001
68	**Greenland**	2.691	0.104	0.093	0.114	<0.001	−0.371	−0.026	−0.049	−0.003	0.031	1.222	0.049	0.038	0.060	<0.001
69	**Grenada**	5.659	0.249	0.194	0.304	<0.001	0.707	0.063	−0.059	0.186	0.299	4.733	0.187	0.168	0.206	<0.001
70	**Guam**	−5.518	−0.258	−0.306	−0.209	<0.001	−2.170	−0.110	−0.137	−0.082	<0.001	−9.831	−0.462	−0.513	−0.410	<0.001
71	**Guatemala**	3.224	0.137	0.106	0.168	<0.001	4.964	0.200	0.172	0.228	<0.001	3.287	0.140	0.113	0.166	<0.001
72	**Guinea**	3.424	0.147	0.111	0.183	<0.001	3.898	0.154	0.116	0.191	<0.001	2.672	0.120	0.088	0.152	<0.001
73	**Guinea-Bissau**	−0.253	−0.019	−0.048	0.010	0.196	−0.373	−0.033	−0.054	−0.013	0.003	1.255	0.044	0.010	0.077	0.012
74	**Guyana**	6.636	0.248	0.230	0.266	<0.001	8.053	0.307	0.281	0.334	<0.001	5.779	0.206	0.203	0.208	<0.001
75	**Haiti**	5.985	0.243	0.218	0.267	<0.001	7.402	0.296	0.262	0.329	<0.001	5.623	0.236	0.208	0.263	<0.001
76	**Honduras**	4.862	0.156	0.145	0.167	<0.001	5.446	0.168	0.155	0.182	<0.001	4.933	0.176	0.171	0.180	<0.001
77	**Hungary**	−16.069	−0.543	−0.615	−0.471	<0.001	−17.486	−0.590	−0.665	−0.514	<0.001	−15.098	−0.512	−0.580	−0.445	<0.001
78	**Iceland**	−5.340	−0.314	−0.385	−0.243	<0.001	−8.058	−0.461	−0.559	−0.363	<0.001	−1.580	−0.098	−0.128	−0.068	<0.001
79	**India**	14.709	0.638	0.534	0.742	<0.001	14.550	0.603	0.519	0.687	<0.001	17.276	0.775	0.641	0.908	<0.001
80	**Indonesia**	−16.520	−0.900	−1.050	−0.749	<0.001	−19.533	−1.044	−1.197	−0.891	<0.001	−10.651	−0.628	−0.772	−0.485	<0.001
81	**Iran**	3.079	0.019	−0.116	0.155	0.772	3.550	0.045	−0.101	0.191	0.534	3.182	0.030	−0.092	0.151	0.617
82	**Iraq**	−0.311	−0.002	−0.013	0.009	0.703	−0.717	−0.017	−0.031	−0.002	0.024	−0.374	−0.003	−0.015	0.009	0.587
83	**Ireland**	2.518	0.068	0.053	0.083	<0.001	1.935	0.041	0.019	0.063	0.001	2.448	0.055	0.042	0.069	<0.001
84	**Israel**	3.152	0.088	0.076	0.100	<0.001	2.727	0.067	0.053	0.081	<0.001	2.628	0.061	0.049	0.073	<0.001
85	**Italy**	−13.545	−0.665	−0.861	−0.469	<0.001	−17.931	−0.893	−1.026	−0.760	<0.001	−8.570	−0.400	−0.714	−0.086	0.015
86	**Jamaica**	6.283	0.244	0.236	0.252	<0.001	7.186	0.265	0.258	0.272	<0.001	4.290	0.177	0.161	0.193	<0.001
87	**Japan**	−1.348	−0.108	−0.141	−0.074	<0.001	−1.209	−0.088	−0.112	−0.064	<0.001	−3.429	−0.209	−0.262	−0.156	<0.001
88	**Jordan**	2.986	0.127	0.100	0.154	<0.001	2.265	0.100	0.089	0.111	<0.001	1.462	0.060	0.045	0.075	<0.001
89	**Kazakhstan**	−8.764	−0.328	−0.360	−0.297	<0.001	−8.258	−0.256	−0.298	−0.213	<0.001	−11.232	−0.480	−0.521	−0.439	<0.001
90	**Kenya**	3.821	0.143	0.110	0.176	<0.001	5.110	0.182	0.155	0.210	<0.001	3.298	0.126	0.090	0.162	<0.001
91	**Kiribati**	−0.710	−0.024	−0.053	0.004	0.084	−0.320	−0.001	−0.040	0.038	0.966	0.481	0.008	−0.001	0.016	0.083
92	**Kuwait**	−1.278	−0.064	−0.096	−0.032	<0.001	1.975	0.080	0.072	0.088	<0.001	1.540	0.069	0.064	0.073	<0.001
93	**Kyrgyzstan**	−3.843	−0.104	−0.159	−0.049	0.001	−7.849	−0.280	−0.338	−0.222	<0.001	0.452	0.096	0.021	0.172	0.015
94	**Laos**	6.147	0.237	0.192	0.282	<0.001	6.120	0.242	0.181	0.303	<0.001	4.152	0.154	0.129	0.180	<0.001
95	**Latvia**	−8.826	−0.395	−0.497	−0.294	<0.001	−9.813	−0.484	−0.576	−0.392	<0.001	−10.211	−0.427	−0.550	−0.304	<0.001
96	**Lebanon**	2.910	0.116	0.104	0.128	<0.001	2.385	0.098	0.087	0.109	<0.001	1.853	0.077	0.065	0.088	<0.001
97	**Lesotho**	3.328	0.129	0.102	0.156	<0.001	4.882	0.186	0.159	0.212	<0.001	3.377	0.136	0.106	0.166	<0.001
98	**Liberia**	2.544	0.153	0.105	0.200	<0.001	3.396	0.186	0.136	0.237	<0.001	3.004	0.162	0.123	0.200	<0.001
99	**Libya**	−0.126	0.032	0.011	0.053	0.004	1.969	0.083	0.074	0.092	<0.001	1.688	0.071	0.062	0.081	<0.001
100	**Lithuania**	−10.033	−0.408	−0.512	−0.304	<0.001	−11.906	−0.480	−0.556	−0.404	<0.001	−7.838	−0.304	−0.427	−0.182	<0.001
101	**Luxembourg**	10.205	0.182	−0.066	0.430	0.144	6.913	0.025	−0.218	0.268	0.835	10.308	0.276	0.010	0.542	0.043
102	**Macedonia**	1.776	0.073	0.057	0.089	<0.001	2.013	0.084	0.071	0.098	<0.001	1.728	0.065	0.053	0.077	<0.001
103	**Madagascar**	2.832	0.118	0.072	0.165	<0.001	4.177	0.161	0.111	0.211	<0.001	2.615	0.121	0.083	0.160	<0.001
104	**Malawi**	3.150	0.111	0.079	0.144	<0.001	4.413	0.159	0.121	0.197	<0.001	3.530	0.149	0.108	0.190	<0.001
105	**Malaysia**	5.510	0.189	0.168	0.211	<0.001	4.894	0.170	0.140	0.201	<0.001	4.000	0.126	0.113	0.138	<0.001
106	**Maldives**	5.354	0.219	0.144	0.295	<0.001	5.877	0.235	0.195	0.276	<0.001	2.305	0.096	0.072	0.121	<0.001
107	**Mali**	3.341	0.144	0.111	0.177	<0.001	3.259	0.136	0.098	0.173	<0.001	2.476	0.121	0.088	0.154	<0.001
108	**Malta**	11.694	0.634	0.494	0.774	<0.001	8.636	0.548	0.389	0.708	<0.001	11.539	0.562	0.462	0.661	<0.001
109	**Marshall Islands**	−3.624	−0.187	−0.209	−0.164	<0.001	−1.073	−0.051	−0.083	−0.020	0.002	−7.890	−0.408	−0.498	−0.318	<0.001
110	**Mauritania**	−0.273	−0.008	−0.037	0.021	0.592	0.142	0.006	−0.032	0.044	0.748	−1.533	−0.049	−0.074	−0.025	<0.001
111	**Mauritius**	3.926	0.128	0.112	0.144	<0.001	3.850	0.122	0.108	0.136	<0.001	3.107	0.100	0.083	0.117	<0.001
112	**Mexico**	−4.399	−0.156	−0.222	−0.090	<0.001	−4.791	−0.200	−0.293	−0.108	<0.001	−3.403	−0.076	−0.115	−0.037	<0.001
113	**Moldova**	5.635	0.286	0.161	0.411	<0.001	5.921	0.297	0.158	0.437	<0.001	4.767	0.257	0.140	0.374	<0.001
114	**Mongolia**	−0.587	0.019	−0.062	0.100	0.637	5.854	0.312	0.220	0.404	<0.001	−6.690	−0.297	−0.375	−0.219	<0.001
115	**Montenegro**	2.168	0.088	0.074	0.101	<0.001	2.149	0.088	0.072	0.105	<0.001	1.444	0.063	0.052	0.075	<0.001
116	**Morocco**	2.288	0.089	0.066	0.112	<0.001	2.144	0.090	0.075	0.104	<0.001	1.932	0.069	0.056	0.082	<0.001
117	**Mozambique**	4.829	0.216	0.190	0.242	<0.001	6.813	0.284	0.264	0.304	<0.001	4.307	0.193	0.159	0.228	<0.001
118	**Myanmar**	4.117	0.176	0.137	0.215	<0.001	6.740	0.283	0.233	0.332	<0.001	4.326	0.170	0.140	0.199	<0.001
119	**Namibia**	1.929	0.094	0.071	0.116	<0.001	2.887	0.117	0.096	0.139	<0.001	2.259	0.117	0.091	0.143	<0.001
120	**Nepal**	8.460	0.321	0.287	0.354	<0.001	11.222	0.415	0.365	0.465	<0.001	7.660	0.285	0.257	0.313	<0.001
121	**Netherlands**	−2.467	−0.098	−0.123	−0.074	<0.001	−4.608	−0.179	−0.205	−0.152	<0.001	−2.554	−0.106	−0.127	−0.085	<0.001
122	**New Zealand**	−21.001	−0.673	−0.915	−0.430	<0.001	−21.187	−0.693	−0.903	−0.482	<0.001	−21.354	−0.674	−0.984	−0.364	<0.001
123	**Nicaragua**	4.133	0.161	0.135	0.186	<0.001	4.729	0.184	0.156	0.211	<0.001	3.051	0.126	0.103	0.148	<0.001
124	**Niger**	1.941	0.097	0.055	0.139	<0.001	2.650	0.117	0.071	0.163	<0.001	2.833	0.146	0.107	0.186	<0.001
125	**Nigeria**	1.185	0.064	0.045	0.083	<0.001	3.089	0.134	0.097	0.170	<0.001	2.587	0.136	0.107	0.166	<0.001
126	**North Korea**	6.919	0.294	0.243	0.346	<0.001	5.019	0.249	0.184	0.314	<0.001	3.930	0.161	0.136	0.185	<0.001
127	**Northern Mariana Islands**	−8.478	−0.340	−0.372	−0.309	<0.001	−0.985	−0.047	−0.071	−0.022	0.001	−15.525	−0.683	−0.706	−0.660	<0.001
128	**Norway**	9.477	0.515	0.412	0.619	<0.001	13.009	0.731	0.562	0.899	<0.001	−3.009	−0.138	−0.162	−0.115	<0.001
129	**Oman**	4.436	0.193	0.123	0.264	<0.001	2.002	0.084	0.075	0.093	<0.001	1.755	0.074	0.068	0.080	<0.001
130	**Pakistan**	8.496	0.316	0.289	0.344	<0.001	10.865	0.389	0.355	0.422	<0.001	6.605	0.260	0.235	0.284	<0.001
131	**Palestine**	4.429	0.156	0.147	0.166	<0.001	2.835	0.114	0.104	0.125	<0.001	2.512	0.099	0.081	0.117	<0.001
132	**Panama**	2.655	0.102	0.078	0.126	<0.001	2.990	0.112	0.087	0.137	<0.001	2.965	0.118	0.096	0.140	<0.001
133	**Papua New Guinea**	0.270	0.005	−0.026	0.037	0.727	2.446	0.089	0.055	0.123	<0.001	−3.156	−0.147	−0.175	−0.119	<0.001
134	**Paraguay**	3.015	0.118	0.110	0.126	<0.001	2.968	0.120	0.108	0.133	<0.001	3.077	0.122	0.114	0.130	<0.001
135	**Peru**	3.332	0.141	0.120	0.161	<0.001	3.272	0.135	0.110	0.161	<0.001	3.377	0.143	0.127	0.160	<0.001
136	**Philippines**	3.481	−0.019	−0.367	0.330	0.911	2.955	−0.060	−0.442	0.323	0.750	6.341	0.144	−0.140	0.428	0.308
137	**Poland**	−6.833	−0.193	−0.223	−0.163	<0.001	−9.146	−0.273	−0.338	−0.208	<0.001	−4.978	−0.139	−0.198	−0.080	<0.001
138	**Portugal**	−1.138	−0.114	−0.156	−0.073	<0.001	−5.605	−0.342	−0.426	−0.258	<0.001	5.214	0.211	0.176	0.247	<0.001
139	**Puerto Rico**	4.651	0.186	0.167	0.206	<0.001	5.721	0.231	0.210	0.251	<0.001	4.282	0.164	0.152	0.175	<0.001
140	**Qatar**	4.731	0.248	0.214	0.282	<0.001	1.720	0.081	0.076	0.087	<0.001	1.492	0.049	0.046	0.051	<0.001
141	**Romania**	1.995	0.088	0.075	0.102	<0.001	1.982	0.089	0.078	0.100	<0.001	1.776	0.074	0.062	0.085	<0.001
142	**Russian Federation**	−8.925	−0.360	−0.462	−0.257	<0.001	−10.881	−0.449	−0.555	−0.343	<0.001	−10.095	−0.383	−0.504	−0.262	<0.001
143	**Rwanda**	2.390	0.121	0.064	0.179	<0.001	3.382	0.149	0.094	0.205	<0.001	3.113	0.152	0.109	0.196	<0.001
144	**Saint Lucia**	6.169	0.229	0.221	0.238	<0.001	6.504	0.260	0.234	0.285	<0.001	3.317	0.110	0.066	0.155	<0.001
145	**Saint Vincent and the Grenadines**	6.514	0.241	0.227	0.255	<0.001	3.842	0.137	0.119	0.154	<0.001	4.552	0.183	0.169	0.197	<0.001
146	**Samoa**	−0.597	−0.049	−0.066	−0.031	<0.001	1.906	0.068	0.046	0.090	<0.001	−4.237	−0.229	−0.251	−0.206	<0.001
147	**Sao Tome and Principe**	3.755	0.167	0.133	0.201	<0.001	3.768	0.165	0.132	0.199	<0.001	2.984	0.138	0.110	0.166	<0.001
148	**Saudi Arabia**	1.690	0.079	0.059	0.100	<0.001	1.999	0.088	0.080	0.096	<0.001	1.981	0.073	0.063	0.084	<0.001
149	**Senegal**	1.543	0.067	0.032	0.102	0.001	1.471	0.050	0.011	0.089	0.014	2.566	0.119	0.088	0.150	<0.001
150	**Serbia**	−0.273	−0.020	−0.043	0.002	0.077	−4.874	−0.264	−0.328	−0.201	<0.001	5.046	0.251	0.215	0.288	<0.001
151	**Seychelles**	5.307	0.186	0.159	0.214	<0.001	3.868	0.117	0.096	0.137	<0.001	3.272	0.112	0.099	0.124	<0.001
152	**Sierra Leone**	2.899	0.125	0.079	0.171	<0.001	2.973	0.115	0.073	0.158	<0.001	2.857	0.134	0.098	0.170	<0.001
153	**Singapore**	−1.730	−0.049	−0.080	−0.018	0.003	−1.903	−0.048	−0.083	−0.013	0.010	−0.040	0.002	−0.023	0.027	0.894
154	**Slovakia**	−3.070	−0.224	−0.272	−0.176	<0.001	1.384	−0.057	−0.140	0.027	0.178	−9.854	−0.512	−0.564	−0.459	<0.001
155	**Slovenia**	−7.616	−0.382	−0.470	−0.294	<0.001	−8.886	−0.439	−0.536	−0.342	<0.001	−8.851	−0.432	−0.509	−0.356	<0.001
156	**Solomon Islands**	−1.853	−0.082	−0.115	−0.048	<0.001	2.609	0.108	0.068	0.147	<0.001	−5.460	−0.260	−0.289	−0.230	<0.001
157	**Somalia**	2.097	0.085	0.036	0.135	0.002	1.969	0.062	0.016	0.108	0.010	3.425	0.157	0.106	0.207	<0.001
158	**South Africa**	3.120	0.142	0.123	0.162	<0.001	3.408	0.143	0.132	0.153	<0.001	2.860	0.138	0.120	0.157	<0.001
159	**South Korea**	3.072	0.095	0.075	0.115	<0.001	1.573	0.026	0.012	0.040	0.001	2.073	0.082	0.067	0.097	<0.001
160	**South Sudan**	−2.323	−0.095	−0.106	−0.083	<0.001	−1.567	−0.061	−0.071	−0.051	<0.001	−0.358	−0.003	−0.028	0.022	0.807
161	**Spain**	3.496	0.106	0.093	0.119	<0.001	2.607	0.071	0.048	0.094	<0.001	2.779	0.071	0.059	0.084	<0.001
162	**Sri Lanka**	0.963	0.030	−0.007	0.067	0.104	3.962	0.145	0.113	0.178	<0.001	2.776	0.091	0.071	0.111	<0.001
163	**Sudan**	2.728	0.110	0.101	0.118	<0.001	2.480	0.106	0.091	0.121	<0.001	2.177	0.091	0.074	0.108	<0.001
164	**Suriname**	1.372	0.110	0.041	0.179	0.003	0.124	0.104	−0.016	0.224	0.086	4.912	0.197	0.183	0.212	<0.001
165	**Swaziland**	2.746	0.107	0.097	0.118	<0.001	3.137	0.114	0.108	0.121	<0.001	3.013	0.123	0.104	0.143	<0.001
166	**Sweden**	−2.233	−0.071	−0.237	0.094	0.384	−5.924	−0.176	−0.383	0.031	0.093	1.160	−0.002	−0.101	0.097	0.967
167	**Switzerland**	7.571	0.149	0.021	0.276	0.024	8.179	0.163	0.014	0.312	0.034	2.701	−0.015	−0.108	0.079	0.749
168	**Syria**	2.343	0.108	0.097	0.119	<0.001	2.499	0.112	0.100	0.124	<0.001	2.447	0.098	0.086	0.110	<0.001
169	**Taiwan**	50.579	1.208	0.738	1.681	<0.001	63.051	1.482	0.937	2.029	<0.001	36.035	0.908	0.585	1.232	<0.001
170	**Tajikistan**	3.941	0.222	0.130	0.314	<0.001	2.512	0.177	0.057	0.296	0.005	3.401	0.188	0.120	0.257	<0.001
171	**Tanzania**	3.344	0.143	0.102	0.184	<0.001	3.792	0.150	0.105	0.194	<0.001	3.318	0.151	0.113	0.188	<0.001
172	**Thailand**	−7.106	−0.369	−0.417	−0.320	<0.001	−11.754	−0.606	−0.695	−0.517	<0.001	2.379	0.053	0.015	0.091	0.009
173	**The Bahamas**	4.776	0.203	0.183	0.224	<0.001	5.729	0.225	0.209	0.241	<0.001	2.392	0.126	0.105	0.146	<0.001
174	**The Gambia**	0.712	0.036	−0.001	0.073	0.056	1.403	0.057	0.024	0.091	0.001	2.278	0.110	0.078	0.142	<0.001
175	**Timor-Leste**	5.936	0.226	0.181	0.271	<0.001	7.670	0.281	0.228	0.335	<0.001	4.081	0.161	0.133	0.189	<0.001
176	**Togo**	1.686	0.072	0.039	0.105	<0.001	2.294	0.086	0.057	0.116	<0.001	2.818	0.133	0.099	0.167	<0.001
177	**Tonga**	1.981	0.065	0.048	0.083	<0.001	1.507	0.046	0.024	0.068	<0.001	1.879	0.068	0.047	0.089	<0.001
178	**Trinidad and Tobago**	1.874	0.169	0.098	0.239	<0.001	0.735	0.129	0.060	0.199	0.001	2.794	0.203	0.137	0.270	<0.001
179	**Tunisia**	1.239	0.053	0.038	0.067	<0.001	2.256	0.091	0.078	0.104	<0.001	1.538	0.069	0.057	0.081	<0.001
180	**Turkey**	7.071	0.407	0.331	0.482	<0.001	11.530	0.613	0.498	0.728	<0.001	−0.535	0.015	−0.009	0.039	0.205
181	**Turkmenistan**	6.083	0.302	0.212	0.391	<0.001	5.477	0.292	0.182	0.403	<0.001	3.725	0.193	0.132	0.255	<0.001
182	**Uganda**	3.251	0.142	0.101	0.183	<0.001	5.502	0.225	0.182	0.267	<0.001	3.935	0.166	0.128	0.203	<0.001
183	**Ukraine**	−6.342	−0.353	−0.523	−0.182	<0.001	−2.580	−0.212	−0.382	−0.042	0.016	−12.187	−0.597	−0.790	−0.404	<0.001
184	**United Arab Emirates**	3.938	0.168	0.147	0.190	<0.001	2.124	0.094	0.087	0.101	<0.001	1.717	0.061	0.055	0.067	<0.001
185	**United Kingdom**	4.643	0.747	0.508	0.987	<0.001	1.631	0.551	0.356	0.747	<0.001	6.956	0.974	0.657	1.293	<0.001
186	**United States**	−5.819	−0.352	−0.559	−0.145	0.002	−7.233	−0.646	−0.877	−0.415	<0.001	−6.156	−0.007	−0.238	0.224	0.949
187	**Uruguay**	2.431	0.088	0.080	0.096	<0.001	3.258	0.120	0.116	0.124	<0.001	1.927	0.068	0.058	0.078	<0.001
188	**Uzbekistan**	4.127	0.228	0.157	0.300	<0.001	4.048	0.237	0.142	0.332	<0.001	2.801	0.163	0.108	0.219	<0.001
189	**Vanuatu**	0.745	0.034	−0.001	0.069	0.058	2.526	0.104	0.076	0.131	<0.001	0.375	0.021	−0.012	0.054	0.207
190	**Venezuela**	1.662	0.067	0.041	0.093	<0.001	2.580	0.089	0.064	0.114	<0.001	0.355	0.024	−0.002	0.051	0.073
191	**Vietnam**	7.532	0.299	0.259	0.338	<0.001	7.540	0.309	0.257	0.361	<0.001	4.169	0.158	0.134	0.183	<0.001
192	**Virgin Islands, U.S.**	1.292	0.065	−0.021	0.151	0.134	6.349	0.246	0.118	0.375	0.001	−3.586	−0.132	−0.172	−0.092	<0.001
193	**Yemen**	2.381	0.089	0.080	0.098	<0.001	2.507	0.102	0.089	0.115	<0.001	2.189	0.088	0.073	0.103	<0.001
194	**Zambia**	−0.354	−0.019	−0.050	0.012	0.214	0.639	0.023	0.007	0.038	0.007	0.674	0.035	−0.013	0.083	0.146
195	**Zimbabwe**	1.296	0.032	0.021	0.043	<0.001	2.546	0.062	0.045	0.079	<0.001	2.560	0.101	0.075	0.127	<0.001

Abbreviations: PC: percentage change; APC: annual percentage change. *p* < 0.001 considered significant.

**Table 4:** Percentage change and annual percentage change of age standardized incidence rate of urolithiasis stratified by gender and SDI level.

	Both Male and Female					Male					Female				
	PC	APC	95%CI	95%CI	*p*-Value	PC	APC	95%CI	95%CI	*p*-Value	PC	APC	95%CI	95%CI	*p*-Value
**Global**	−9.995	−0.459	−0.506	−0.411	<0.001	−11.433	−0.557	−0.615	−0.499	<0.001	−8.227	−0.312	−0.367	−0.258	<0.001
**Low SDI**	7.620	0.335	0.268	0.403	<0.001	9.028	0.375	0.307	0.443	<0.001	7.492	0.352	0.280	0.423	<0.001
**Low-middle SDI**	2.899	0.121	0.094	0.147	<0.001	3.145	0.107	0.089	0.126	<0.001	4.540	0.221	0.174	0.268	<0.001
**Middle SDI**	−11.840	−0.625	−0.731	−0.520	<0.001	−14.600	−0.783	−0.920	−0.646	<0.001	−5.982	−0.309	−0.363	−0.254	<0.001
**High-middle SDI**	−23.757	−1.165	−1.255	−1.074	<0.001	−24.098	−1.204	−1.311	−1.096	<0.001	−25.151	−1.195	−1.271	−1.120	<0.001
**High SDI**	−1.774	−0.103	−0.174	−0.033	0.006	−3.155	−0.218	−0.293	−0.143	<0.001	−2.358	−0.009	−0.096	0.079	0.840

Abbreviations: PC: percentage change; APC: annual percentage change; SDI: sociodemographic index. *p* < 0.001 considered significant.

**Table 5:** Percentage change and annual percentage change of disability adjusted life years of urolithiasis stratified by gender and 21 regions.

	Both Male and Female					Male					Female				
	PC	APC	95%CI	95%CI	*p*-Value	PC	APC	95%CI	95%CI	*p*-Value	PC	APC	95%CI	95%CI	*p*-Value
**Central Asia**	−18.427	−1.325	−1.663	−0.986	<0.001	−15.861	−1.145	−1.439	−0.849	<0.001	−23.294	−1.646	−2.061	−1.230	<0.001
**Eastern Asia**	−65.808	−4.678	−4.953	−4.403	<0.001	−62.349	−4.110	−4.352	−3.867	<0.001	−71.220	−5.633	−6.046	−5.218	<0.001
**High-income Asia Pacific**	13.003	0.670	0.577	0.762	<0.001	4.323	0.270	0.200	0.341	<0.001	22.671	1.092	0.964	1.221	<0.001
**South Asia**	−11.841	−0.459	−0.562	−0.355	<0.001	−10.493	−0.343	−0.425	−0.261	<0.001	−10.347	−0.477	−0.659	−0.295	<0.001
**Southeast Asia**	3.713	−0.284	−0.517	−0.050	0.019	−5.895	−0.742	−0.989	−0.494	<0.001	30.817	0.765	0.470	1.060	<0.001
**Central Europe**	−53.614	−2.776	−3.305	−2.245	<0.001	−52.706	−2.678	−3.177	−2.176	<0.001	−55.245	−2.947	−3.509	−2.382	<0.001
**Eastern Europe**	−28.217	−1.768	−2.177	−1.357	<0.001	−29.962	−1.845	−2.239	−1.451	<0.001	−30.651	−1.918	−2.369	−1.465	<0.001
**Western Europe**	−10.909	−0.137	−0.318	0.044	0.132	−11.504	−0.168	−0.346	0.010	0.063	−12.669	−0.199	−0.398	<0.001	0.050
**Andean Latin America**	−10.561	−0.323	−0.406	−0.240	<0.001	−12.678	−0.359	−0.442	−0.276	<0.001	−7.983	−0.280	−0.383	−0.176	<0.001
**Central Latin America**	3.083	0.172	−0.127	0.471	0.249	−4.440	−0.057	−0.328	0.214	0.668	12.273	0.420	0.082	0.760	0.017
**Southern Latin America**	−2.953	−0.181	−0.229	−0.134	<0.001	−2.691	−0.129	−0.206	−0.052	0.002	−3.069	−0.226	−0.296	−0.156	<0.001
**Tropical Latin America**	115.258	3.248	3.142	3.354	<0.001	89.802	2.770	2.649	2.892	<0.001	141.624	3.668	3.549	3.788	<0.001
**High-income North America**	−2.392	0.022	−0.168	0.212	0.815	−7.531	−0.391	−0.584	−0.197	<0.001	1.858	0.454	0.223	0.687	<0.001
**Central Sub-Saharan Africa**	−3.961	−0.254	−0.318	−0.190	<0.001	−1.286	−0.165	−0.228	−0.101	<0.001	−4.459	−0.275	−0.336	−0.214	<0.001
**Eastern Sub-Saharan Africa**	−21.744	−1.194	−1.319	−1.069	<0.001	−19.306	−1.049	−1.160	−0.938	<0.001	−23.094	−1.288	−1.440	−1.137	<0.001
**Southern Sub-Saharan Africa**	−2.270	−0.264	−0.789	0.263	0.312	5.779	−0.005	−0.523	0.516	0.984	−11.691	−0.614	−1.195	−0.029	0.040
**Western Sub-Saharan Africa**	−17.285	−0.993	−1.146	−0.839	<0.001	−15.489	−0.978	−1.166	−0.790	<0.001	−19.677	−1.002	−1.119	−0.885	<0.001
**North Africa and Middle East**	0.932	0.035	−0.036	0.106	0.322	3.794	0.206	0.143	0.268	<0.001	−4.249	−0.274	−0.381	−0.167	<0.001
**Oceania**	−29.103	−1.420	−1.555	−1.284	<0.001	−22.757	−1.021	−1.195	−0.848	<0.001	−33.942	−1.724	−1.837	−1.611	<0.001
**Australasia**	−34.669	−1.304	−1.686	−0.920	<0.001	−37.597	−1.509	−1.890	−1.127	<0.001	−33.330	−1.185	−1.584	−0.784	<0.001
**Caribbean**	25.854	1.133	0.986	1.280	<0.001	38.205	1.401	1.284	1.518	<0.001	14.286	0.832	0.572	1.093	<0.001

Abbreviations: PC: percentage change; APC: annual percentage change. *p* < 0.001 considered significant.

**Table 6:** Percentage change and annual percentage change of disability adjusted life years of urolithiasis stratified by gender and 195 Countries and territories.

		Both Male and Female					Male					Female				
Number	Countries and Territories	PC	APC	95%CI	95%CI	*p*-Value	PC	APC	95%CI	95%CI	*p*-Value	PC	APC	95%CI	95%CI	*p*-Value
1	**Afghanistan**	15.058	0.712	0.610	0.813	<0.001	12.701	0.588	0.494	0.682	<0.001	20.151	0.927	0.809	1.046	<0.001
2	**Albania**	−23.995	−0.891	−1.161	−0.620	<0.001	−37.147	−1.568	−2.023	−1.110	<0.001	0.662	−0.016	−0.041	0.008	0.182
3	**Algeria**	5.555	0.249	0.228	0.270	<0.001	3.006	0.132	0.114	0.150	<0.001	9.369	0.422	0.386	0.459	<0.001
4	**American Samoa**	−57.879	−4.974	−6.065	−3.871	<0.001	−63.640	−5.687	−6.894	−4.463	<0.001	−49.481	−4.080	−5.072	−3.078	<0.001
5	**Andorra**	−8.383	−0.309	−0.421	−0.198	<0.001	−10.128	−0.345	−0.413	−0.276	<0.001	−4.363	−0.158	−0.361	0.045	0.121
6	**Angola**	−6.882	−0.471	−0.562	−0.381	<0.001	−3.606	−0.353	−0.449	−0.256	<0.001	−5.742	−0.409	−0.497	−0.320	<0.001
7	**Antigua and Barbuda**	28.971	1.047	0.839	1.256	<0.001	9.861	0.373	0.262	0.485	<0.001	44.867	1.578	1.246	1.910	<0.001
8	**Argentina**	−1.901	−0.007	−0.112	0.097	0.889	−5.406	−0.163	−0.320	−0.005	0.044	1.871	0.151	0.083	0.218	<0.001
9	**Armenia**	50.908	1.995	1.279	2.716	<0.001	61.061	2.221	1.585	2.860	<0.001	39.743	1.669	0.877	2.467	<0.001
10	**Australia**	−35.315	−1.425	−1.752	−1.098	<0.001	−39.554	−1.654	−1.993	−1.313	<0.001	−32.122	−1.275	−1.602	−0.946	<0.001
11	**Austria**	−50.089	−1.786	−2.365	−1.203	<0.001	−40.020	−0.955	−1.356	−0.552	<0.001	−59.048	−2.804	−3.593	−2.007	<0.001
12	**Azerbaijan**	23.858	0.821	0.638	1.003	<0.001	10.600	0.414	0.373	0.454	<0.001	32.914	1.083	0.770	1.396	<0.001
13	**Bahrain**	5.745	−0.134	−0.361	0.093	0.235	4.565	0.143	0.101	0.186	<0.001	2.802	−0.805	−1.339	−0.268	0.005
14	**Bangladesh**	−16.943	−0.491	−0.615	−0.367	<0.001	−6.171	0.045	−0.129	0.218	0.601	−29.580	−1.239	−1.341	−1.138	<0.001
15	**Barbados**	−4.192	0.201	0.055	0.347	0.009	−4.387	0.078	−0.053	0.209	0.234	−6.469	0.252	−0.079	0.584	0.130
16	**Belarus**	−31.658	−1.956	−2.133	−1.779	<0.001	−20.341	−1.298	−1.449	−1.147	<0.001	−41.282	−2.606	−2.816	−2.396	<0.001
17	**Belgium**	28.074	1.353	1.041	1.665	<0.001	27.147	1.251	0.944	1.559	<0.001	27.233	1.404	1.086	1.722	<0.001
18	**Belize**	41.679	1.259	1.023	1.495	<0.001	47.698	1.181	0.892	1.472	<0.001	35.897	1.269	1.012	1.526	<0.001
19	**Benin**	−17.242	−1.019	−1.178	−0.860	<0.001	−20.757	−1.373	−1.610	−1.136	<0.001	−9.717	−0.421	−0.494	−0.347	<0.001
20	**Bermuda**	−6.617	−0.057	−0.235	0.122	0.521	1.328	0.316	0.232	0.401	<0.001	−12.465	−0.349	−0.643	−0.054	0.022
21	**Bhutan**	−20.917	−0.897	−0.931	−0.863	<0.001	−19.047	−0.699	−0.762	−0.635	<0.001	−24.403	−1.193	−1.257	−1.129	<0.001
22	**Bolivia**	−21.064	−0.619	−0.781	−0.457	<0.001	−23.808	−0.697	−0.856	−0.538	<0.001	−17.851	−0.523	−0.699	−0.347	<0.001
23	**Bosnia and Herzegovina**	−22.795	−1.381	−1.636	−1.126	<0.001	−23.227	−1.377	−1.639	−1.114	<0.001	−24.058	−1.500	−1.763	−1.237	<0.001
24	**Botswana**	−3.975	−0.072	−0.220	0.075	0.321	−17.143	−0.848	−0.910	−0.786	<0.001	15.300	0.932	0.655	1.209	<0.001
25	**Brazil**	116.654	3.279	3.170	3.387	<0.001	91.467	2.815	2.689	2.941	<0.001	142.686	3.686	3.565	3.808	<0.001
26	**Brunei**	−23.121	−1.015	−1.279	−0.750	<0.001	−24.840	−1.126	−1.421	−0.830	<0.001	0.425	0.020	−0.050	0.089	0.564
27	**Bulgaria**	−75.459	−6.073	−7.056	−5.080	<0.001	−76.813	−6.330	−7.323	−5.326	<0.001	−73.777	−5.784	−6.760	−4.799	<0.001
28	**Burkina Faso**	−13.408	−0.692	−0.827	−0.557	<0.001	−14.846	−0.880	−1.083	−0.676	<0.001	−8.763	−0.328	−0.386	−0.271	<0.001
29	**Burundi**	−24.946	−1.398	−1.569	−1.228	<0.001	−29.735	−1.651	−1.828	−1.474	<0.001	−25.138	−1.403	−1.575	−1.231	<0.001
30	**Cambodia**	−23.372	−0.995	−1.090	−0.901	<0.001	−21.376	−0.830	−0.945	−0.715	<0.001	−24.158	−1.160	−1.245	−1.075	<0.001
31	**Cameroon**	−22.199	−1.383	−1.593	−1.172	<0.001	−14.109	−1.106	−1.354	−0.857	<0.001	−30.550	−1.724	−1.904	−1.544	<0.001
32	**Canada**	8.418	0.633	0.496	0.771	<0.001	7.003	0.580	0.443	0.718	<0.001	8.398	0.639	0.491	0.788	<0.001
33	**Cape Verde**	6.343	0.233	0.200	0.265	<0.001	5.101	0.147	0.125	0.169	<0.001	5.483	0.237	0.180	0.293	<0.001
34	**Central African Republic**	2.214	0.054	−0.014	0.122	0.115	5.567	0.127	0.045	0.210	0.004	−2.899	−0.073	−0.143	−0.002	0.044
35	**Chad**	−17.860	−1.071	−1.270	−0.872	<0.001	−19.333	−1.272	−1.537	−1.005	<0.001	−19.760	−0.988	−1.125	−0.851	<0.001
36	**Chile**	−12.798	−0.870	−1.069	−0.670	<0.001	−5.288	−0.471	−0.663	−0.279	<0.001	−18.618	−1.207	−1.453	−0.960	<0.001
37	**China**	−66.979	−4.811	−5.084	−4.538	<0.001	−63.542	−4.229	−4.472	−3.985	<0.001	−72.305	−5.788	−6.200	−5.374	<0.001
38	**Colombia**	25.487	1.495	1.064	1.928	<0.001	13.824	1.114	0.713	1.516	<0.001	39.262	1.896	1.419	2.376	<0.001
39	**Comoros**	−15.419	−0.813	−0.969	−0.657	<0.001	−13.760	−0.746	−0.947	−0.545	<0.001	−14.269	−0.760	−0.869	−0.650	<0.001
40	**Congo**	−6.931	−0.410	−0.500	−0.320	<0.001	−11.980	−0.634	−0.748	−0.519	<0.001	−2.571	−0.239	−0.342	−0.136	<0.001
41	**Costa Rica**	43.455	1.843	1.609	2.078	<0.001	13.993	0.591	0.525	0.657	<0.001	81.502	3.173	2.741	3.606	<0.001
42	**Cote d’Ivoire**	−15.270	−0.974	−1.168	−0.780	<0.001	−14.881	−1.092	−1.328	−0.856	<0.001	−14.963	−0.756	−0.937	−0.574	<0.001
43	**Croatia**	−6.659	0.145	−0.264	0.555	0.474	−18.495	−0.620	−1.021	−0.217	0.004	4.232	0.771	0.357	1.187	0.001
44	**Cuba**	26.970	1.134	0.791	1.479	<0.001	47.845	1.511	1.189	1.833	<0.001	10.222	0.729	0.263	1.197	0.003
45	**Cyprus**	−5.585	−0.379	−0.473	−0.286	<0.001	−11.608	−0.702	−0.914	−0.490	<0.001	2.182	0.028	−0.099	0.156	0.651
46	**Czech Republic**	−71.761	−4.669	−5.529	−3.801	<0.001	−73.941	−4.850	−5.775	−3.915	<0.001	−70.806	−4.668	−5.480	−3.849	<0.001
47	**Democratic Republic of the Congo**	−3.776	−0.224	−0.304	−0.144	<0.001	−0.375	−0.101	−0.167	−0.035	0.004	−4.935	−0.286	−0.372	−0.201	<0.001
48	**Denmark**	−2.850	0.091	−0.174	0.357	0.486	11.165	0.555	0.258	0.853	0.001	−19.951	−0.587	−0.903	−0.270	0.001
49	**Djibouti**	0.286	−0.376	−0.521	−0.232	<0.001	−2.195	−0.479	−0.620	−0.338	<0.001	1.441	−0.285	−0.435	−0.134	0.001
50	**Dominica**	40.114	1.326	1.256	1.397	<0.001	37.017	1.210	1.119	1.302	<0.001	33.026	1.136	1.045	1.227	<0.001
51	**Dominican Republic**	1.664	−0.073	−0.337	0.191	0.573	−1.139	−0.212	−0.690	0.269	0.373	6.721	0.111	−0.041	0.264	0.145
52	**Ecuador**	9.434	0.691	0.442	0.941	<0.001	8.115	0.687	0.450	0.925	<0.001	11.862	0.713	0.391	1.036	<0.001
53	**Egypt**	8.930	0.390	0.356	0.424	<0.001	6.212	0.287	0.256	0.319	<0.001	13.391	0.557	0.512	0.602	<0.001
54	**El Salvador**	1.623	−0.119	−0.306	0.069	0.205	−7.075	−0.568	−0.890	−0.244	0.001	15.091	0.440	0.350	0.530	<0.001
55	**Equatorial Guinea**	−10.710	−0.510	−0.651	−0.368	<0.001	−19.258	−1.013	−1.273	−0.752	<0.001	3.719	0.148	0.086	0.210	<0.001
56	**Eritrea**	−15.875	−1.047	−1.186	−0.908	<0.001	−20.252	−1.339	−1.529	−1.148	<0.001	−4.624	−0.374	−0.444	−0.303	<0.001
57	**Estonia**	−61.106	−4.732	−5.227	−4.234	<0.001	−59.551	−4.761	−5.354	−4.164	<0.001	−64.200	−4.875	−5.344	−4.403	<0.001
58	**Ethiopia**	−44.034	−2.491	−2.662	−2.319	<0.001	−47.116	−2.668	−2.873	−2.464	<0.001	−39.946	−2.255	−2.415	−2.096	<0.001
59	**Federated States of Micronesia**	−34.393	−1.723	−1.849	−1.597	<0.001	−26.199	−1.289	−1.410	−1.168	<0.001	−40.328	−2.056	−2.231	−1.881	<0.001
60	**Fiji**	25.048	1.178	0.967	1.390	<0.001	27.600	1.216	1.016	1.416	<0.001	24.654	1.230	0.885	1.576	<0.001
61	**Finland**	−11.289	−0.444	−0.667	−0.221	<0.001	1.896	0.212	0.036	0.388	0.020	−23.446	−1.133	−1.407	−0.859	<0.001
62	**France**	−12.134	−0.400	−0.458	−0.341	<0.001	−18.534	−0.654	−0.793	−0.514	<0.001	−5.881	−0.179	−0.281	−0.077	0.001
63	**Gabon**	4.662	0.103	−0.018	0.223	0.091	6.645	0.146	0.065	0.228	0.001	−0.965	−0.069	−0.257	0.118	0.454
64	**Georgia**	14.420	0.541	0.272	0.810	<0.001	9.658	0.355	0.126	0.585	0.004	18.756	0.717	0.385	1.050	<0.001
65	**Germany**	1.008	0.469	0.246	0.693	<0.001	8.277	0.650	0.474	0.827	<0.001	−9.405	0.106	−0.187	0.400	0.462
66	**Ghana**	−3.956	−0.350	−0.477	−0.223	<0.001	−1.070	−0.299	−0.457	−0.140	0.001	2.449	0.057	0.009	0.105	0.022
67	**Greece**	1.048	0.050	−0.014	0.114	0.123	0.799	0.019	−0.045	0.084	0.542	0.761	0.073	0.002	0.145	0.046
68	**Greenland**	−6.682	−0.370	−0.476	−0.265	<0.001	−20.369	−1.109	−1.340	−0.878	<0.001	3.067	0.129	0.108	0.149	<0.001
69	**Grenada**	27.548	1.232	1.029	1.435	<0.001	3.555	0.458	0.075	0.843	0.021	83.321	2.577	1.900	3.258	<0.001
70	**Guam**	−45.456	−3.492	−4.224	−2.754	<0.001	−33.399	−2.084	−2.531	−1.634	<0.001	−55.967	−4.844	−5.853	−3.824	<0.001
71	**Guatemala**	−30.566	−2.093	−2.535	−1.650	<0.001	−21.607	−1.362	−1.723	−0.999	<0.001	−37.111	−2.654	−3.181	−2.124	<0.001
72	**Guinea**	−5.443	−0.339	−0.475	−0.204	<0.001	−1.498	−0.231	−0.374	−0.089	0.003	−10.203	−0.488	−0.622	−0.355	<0.001
73	**Guinea-Bissau**	−29.523	−1.596	−1.783	−1.410	<0.001	−29.331	−1.667	−1.878	−1.457	<0.001	−26.518	−1.341	−1.498	−1.183	<0.001
74	**Guyana**	37.366	1.355	1.046	1.665	<0.001	36.173	1.443	1.048	1.839	<0.001	39.750	1.254	0.940	1.569	<0.001
75	**Haiti**	19.893	0.842	0.744	0.940	<0.001	23.367	1.013	0.866	1.160	<0.001	17.824	0.707	0.646	0.768	<0.001
76	**Honduras**	13.799	0.080	−0.198	0.359	0.559	−0.859	−0.265	−0.475	−0.055	0.016	36.640	0.484	0.108	0.861	0.014
77	**Hungary**	−66.925	−4.065	−4.742	−3.382	<0.001	−64.592	−3.837	−4.430	−3.240	<0.001	−68.963	−4.292	−5.042	−3.535	<0.001
78	**Iceland**	−32.983	−1.057	−1.175	−0.939	<0.001	−29.684	−1.017	−1.178	−0.856	<0.001	−37.661	−1.115	−1.280	−0.950	<0.001
79	**India**	−13.762	−0.536	−0.660	−0.412	<0.001	−13.901	−0.475	−0.571	−0.379	<0.001	−10.155	−0.469	−0.679	−0.259	<0.001
80	**Indonesia**	−39.727	−1.912	−2.058	−1.766	<0.001	−41.372	−2.014	−2.187	−1.840	<0.001	−31.422	−1.414	−1.503	−1.324	<0.001
81	**Iran**	13.478	0.546	0.295	0.797	<0.001	12.096	0.509	0.279	0.740	<0.001	16.618	0.652	0.366	0.939	<0.001
82	**Iraq**	−47.971	−3.087	−3.377	−2.796	<0.001	−43.436	−2.759	−3.018	−2.499	<0.001	−54.267	−3.554	−3.911	−3.195	<0.001
83	**Ireland**	−15.086	−0.565	−0.761	−0.370	<0.001	−26.184	−1.091	−1.453	−0.729	<0.001	4.495	0.130	−0.018	0.278	0.084
84	**Israel**	29.734	0.356	−0.096	0.811	0.118	29.298	0.493	0.115	0.873	0.012	30.053	0.155	−0.457	0.771	0.608
85	**Italy**	−37.806	−1.542	−1.795	−1.288	<0.001	−42.264	−1.914	−2.177	−1.651	<0.001	−33.939	−1.182	−1.454	−0.909	<0.001
86	**Jamaica**	49.334	1.658	1.343	1.973	<0.001	65.546	1.873	1.334	2.414	<0.001	24.847	1.166	0.899	1.434	<0.001
87	**Japan**	18.230	0.921	0.817	1.024	<0.001	10.197	0.579	0.507	0.651	<0.001	27.160	1.295	1.147	1.444	<0.001
88	**Jordan**	−13.413	−0.912	−1.121	−0.703	<0.001	−0.877	−0.029	−0.051	−0.007	0.012	−28.806	−2.061	−2.540	−1.580	<0.001
89	**Kazakhstan**	−48.206	−3.577	−4.094	−3.058	<0.001	−46.692	−3.373	−3.850	−2.893	<0.001	−52.573	−4.008	−4.572	−3.442	<0.001
90	**Kenya**	1.419	−0.086	−0.233	0.061	0.238	12.178	0.355	0.183	0.527	<0.001	−8.524	−0.563	−0.703	−0.422	<0.001
91	**Kiribati**	−14.146	−0.598	−0.670	−0.526	<0.001	−24.167	−0.993	−1.117	−0.870	<0.001	−1.074	−0.196	−0.432	0.040	0.100
92	**Kuwait**	−4.534	−0.061	−0.117	−0.006	0.032	2.149	0.207	0.118	0.297	<0.001	−11.842	−0.360	−0.556	−0.165	0.001
93	**Kyrgyzstan**	−47.085	−2.382	−2.751	−2.012	<0.001	−40.727	−1.931	−2.261	−1.600	<0.001	−58.852	−3.359	−3.874	−2.841	<0.001
94	**Laos**	−32.871	−1.584	−1.701	−1.466	<0.001	−33.167	−1.573	−1.679	−1.466	<0.001	−33.025	−1.653	−1.802	−1.505	<0.001
95	**Latvia**	−41.423	−2.890	−3.303	−2.475	<0.001	−31.099	−2.611	−3.192	−2.027	<0.001	−49.674	−3.279	−3.671	−2.887	<0.001
96	**Lebanon**	8.621	0.396	0.361	0.431	<0.001	6.520	0.323	0.284	0.361	<0.001	10.250	0.457	0.418	0.496	<0.001
97	**Lesotho**	12.904	0.670	0.578	0.763	<0.001	16.230	0.729	0.579	0.879	<0.001	11.289	0.703	0.576	0.831	<0.001
98	**Liberia**	−17.119	−0.828	−1.035	−0.620	<0.001	−18.339	−0.959	−1.170	−0.747	<0.001	−13.551	−0.543	−0.747	−0.339	<0.001
99	**Libya**	17.284	0.741	0.673	0.809	<0.001	11.056	0.444	0.411	0.478	<0.001	33.826	1.332	1.233	1.432	<0.001
100	**Lithuania**	−47.842	−3.349	−3.858	−2.838	<0.001	−48.493	−3.631	−4.157	−3.102	<0.001	−48.086	−3.148	−3.675	−2.618	<0.001
101	**Luxembourg**	−6.809	−0.215	−0.327	−0.103	0.001	−10.999	−0.432	−0.540	−0.323	<0.001	−5.709	−0.077	−0.196	0.043	0.201
102	**Macedonia**	−0.982	−0.008	−0.047	0.031	0.678	−0.244	0.008	−0.030	0.045	0.679	−1.651	−0.021	−0.061	0.019	0.283
103	**Madagascar**	−12.057	−0.596	−0.672	−0.521	<0.001	−7.941	−0.360	−0.415	−0.305	<0.001	−14.583	−0.784	−0.890	−0.678	<0.001
104	**Malawi**	0.153	−0.418	−0.647	−0.189	0.001	14.368	0.144	−0.083	0.372	0.203	−13.592	−1.049	−1.298	−0.798	<0.001
105	**Malaysia**	16.685	0.449	0.339	0.559	<0.001	3.164	0.018	−0.172	0.208	0.847	35.040	0.974	0.783	1.166	<0.001
106	**Maldives**	−40.651	−2.037	−2.250	−1.824	<0.001	−33.213	−1.556	−1.715	−1.396	<0.001	−50.703	−2.815	−3.057	−2.573	<0.001
107	**Mali**	−28.485	−1.442	−1.659	−1.225	<0.001	−26.824	−1.353	−1.602	−1.105	<0.001	−30.630	−1.545	−1.734	−1.355	<0.001
108	**Malta**	−12.206	−0.148	−0.363	0.068	0.170	−14.035	−0.211	−0.385	−0.038	0.019	−12.604	−0.204	−0.494	0.087	0.161
109	**Marshall Islands**	−22.149	−1.361	−1.567	−1.155	<0.001	−32.532	−1.686	−1.822	−1.549	<0.001	−16.266	−1.231	−1.557	−0.905	<0.001
110	**Mauritania**	−35.008	−1.965	−2.156	−1.774	<0.001	−33.062	−1.911	−2.155	−1.666	<0.001	−37.553	−2.064	−2.217	−1.911	<0.001
111	**Mauritius**	−5.955	<0.001	−0.295	0.295	0.999	−14.964	−0.360	−0.704	−0.014	0.042	7.802	0.489	0.188	0.791	0.003
112	**Mexico**	4.368	0.326	−0.043	0.696	0.081	−6.273	−0.036	−0.407	0.336	0.843	17.910	0.713	0.336	1.093	0.001
113	**Moldova**	−6.416	0.074	−0.336	0.486	0.714	−13.379	−0.478	−0.836	−0.120	0.011	1.525	0.685	0.138	1.235	0.016
114	**Mongolia**	−53.422	−4.520	−5.291	−3.743	<0.001	−14.020	−1.099	−1.330	−0.868	<0.001	−69.981	−6.773	−7.858	−5.675	<0.001
115	**Montenegro**	1.741	0.069	0.056	0.082	<0.001	1.878	0.063	0.044	0.082	<0.001	0.881	0.053	0.039	0.068	<0.001
116	**Morocco**	9.513	0.431	0.393	0.468	<0.001	5.685	0.267	0.240	0.294	<0.001	15.483	0.681	0.620	0.742	<0.001
117	**Mozambique**	15.313	0.529	0.411	0.647	<0.001	17.373	0.648	0.535	0.760	<0.001	19.983	0.577	0.433	0.722	<0.001
118	**Myanmar**	−23.528	−0.869	−0.944	−0.793	<0.001	−21.745	−0.787	−0.865	−0.709	<0.001	−22.179	−0.808	−0.886	−0.730	<0.001
119	**Namibia**	−10.046	−0.631	−0.809	−0.452	<0.001	−8.798	−0.583	−0.757	−0.409	<0.001	−8.622	−0.570	−0.757	−0.382	<0.001
120	**Nepal**	−3.278	0.056	−0.262	0.376	0.719	2.461	0.288	−0.028	0.606	0.073	−7.507	−0.145	−0.491	0.202	0.397
121	**Netherlands**	−31.996	−1.599	−1.764	−1.435	<0.001	−28.690	−1.203	−1.321	−1.085	<0.001	−36.399	−2.067	−2.303	−1.829	<0.001
122	**New Zealand**	−31.326	−0.822	−1.421	−0.219	0.009	−27.960	−0.849	−1.436	−0.259	0.007	−35.417	−0.890	−1.535	−0.242	0.009
123	**Nicaragua**	−3.632	−0.228	−0.409	−0.046	0.016	−10.879	−0.767	−0.964	−0.570	<0.001	4.232	0.366	0.061	0.672	0.020
124	**Niger**	−28.859	−1.614	−1.832	−1.395	<0.001	−30.570	−1.758	−2.010	−1.505	<0.001	−26.264	−1.345	−1.518	−1.172	<0.001
125	**Nigeria**	−19.199	−1.143	−1.318	−0.968	<0.001	−17.416	−1.109	−1.307	−0.911	<0.001	−21.918	−1.194	−1.372	−1.015	<0.001
126	**North Korea**	−6.676	−0.287	−0.341	−0.233	<0.001	−9.133	−0.378	−0.424	−0.333	<0.001	−10.659	−0.498	−0.571	−0.424	<0.001
127	**Northern Mariana Islands**	−55.683	−4.148	−4.922	−3.368	<0.001	−16.670	−0.981	−1.260	−0.701	<0.001	−70.243	−5.838	−6.832	−4.833	<0.001
128	**Norway**	−16.129	−0.384	−0.596	−0.172	0.001	−14.935	−0.277	−0.510	−0.042	0.022	−23.330	−0.827	−1.019	−0.634	<0.001
129	**Oman**	4.628	0.205	0.124	0.287	<0.001	2.123	0.101	0.076	0.125	<0.001	2.420	0.103	0.055	0.151	<0.001
130	**Pakistan**	13.588	0.260	0.147	0.374	<0.001	17.216	0.418	0.300	0.537	<0.001	11.256	0.093	−0.023	0.209	0.110
131	**Palestine**	−6.057	−0.206	−0.238	−0.174	<0.001	−3.489	−0.128	−0.150	−0.107	<0.001	−12.683	−0.422	−0.499	−0.344	<0.001
132	**Panama**	9.629	0.448	0.169	0.728	0.003	12.047	0.583	0.423	0.744	<0.001	7.124	0.302	−0.180	0.786	0.209
133	**Papua New Guinea**	−33.681	−1.629	−1.746	−1.512	<0.001	−26.695	−1.206	−1.364	−1.047	<0.001	−38.621	−1.935	−2.016	−1.854	<0.001
134	**Paraguay**	48.563	1.575	1.354	1.797	<0.001	15.725	0.346	0.121	0.572	0.004	88.890	2.749	2.413	3.085	<0.001
135	**Peru**	−16.371	−0.781	−0.953	−0.609	<0.001	−18.986	−0.858	−0.988	−0.728	<0.001	−13.357	−0.700	−0.919	−0.481	<0.001
136	**Philippines**	190.856	1.316	0.107	2.539	0.034	175.645	1.039	−0.179	2.273	0.092	241.267	2.107	0.889	3.340	0.001
137	**Poland**	−47.909	−2.451	−2.856	−2.044	<0.001	−42.002	−2.023	−2.351	−1.693	<0.001	−53.679	−2.958	−3.446	−2.468	<0.001
138	**Portugal**	8.691	0.631	0.335	0.928	<0.001	−5.501	0.033	−0.300	0.368	0.838	27.336	1.307	1.022	1.592	<0.001
139	**Puerto Rico**	23.834	0.761	0.614	0.908	<0.001	29.975	1.209	1.061	1.356	<0.001	18.493	0.333	0.053	0.615	0.022
140	**Qatar**	−0.915	−0.049	−0.074	−0.024	<0.001	−2.979	−0.127	−0.151	−0.102	<0.001	−2.666	−0.322	−0.416	−0.228	<0.001
141	**Romania**	−2.319	−0.084	−0.143	−0.026	0.006	2.075	0.102	0.076	0.127	<0.001	−7.684	−0.329	−0.429	−0.229	<0.001
142	**Russian Federation**	−26.499	−1.568	−2.014	−1.120	<0.001	−33.817	−1.941	−2.383	−1.497	<0.001	−25.272	−1.521	−2.007	−1.032	<0.001
143	**Rwanda**	−27.563	−1.702	−1.942	−1.461	<0.001	−24.589	−1.594	−1.830	−1.358	<0.001	−27.309	−1.609	−1.889	−1.328	<0.001
144	**Saint Lucia**	26.887	0.713	0.503	0.925	<0.001	9.420	0.280	0.221	0.338	<0.001	37.191	0.973	0.707	1.240	<0.001
145	**Saint Vincent and the Grenadines**	10.513	0.744	0.418	1.072	<0.001	2.150	0.533	0.159	0.909	0.007	3.426	0.119	−0.058	0.296	0.179
146	**Samoa**	−29.618	−1.749	−1.933	−1.566	<0.001	−21.364	−1.101	−1.251	−0.952	<0.001	−34.408	−2.152	−2.368	−1.935	<0.001
147	**Sao Tome and Principe**	5.229	0.064	−0.003	0.132	0.061	−1.076	−0.032	−0.134	0.070	0.524	10.786	0.131	<0.001	0.262	0.050
148	**Saudi Arabia**	3.974	0.205	0.184	0.226	<0.001	2.717	0.146	0.122	0.170	<0.001	8.682	0.399	0.365	0.434	<0.001
149	**Senegal**	−24.148	−1.349	−1.573	−1.124	<0.001	−25.833	−1.486	−1.747	−1.225	<0.001	−19.669	−1.084	−1.260	−0.909	<0.001
150	**Serbia**	−1.160	−0.086	−0.146	−0.026	0.007	−10.382	−0.606	−0.731	−0.481	<0.001	8.550	0.417	0.324	0.511	<0.001
151	**Seychelles**	21.264	0.523	0.414	0.632	<0.001	13.555	0.110	−0.063	0.282	0.203	30.352	1.029	0.929	1.129	<0.001
152	**Sierra Leone**	−13.421	−0.749	−0.872	−0.627	<0.001	−18.198	−1.069	−1.242	−0.896	<0.001	−5.769	−0.288	−0.376	−0.200	<0.001
153	**Singapore**	−38.987	−1.374	−1.787	−0.959	<0.001	−30.130	−1.010	−1.523	−0.495	<0.001	−48.980	−1.891	−2.239	−1.542	<0.001
154	**Slovakia**	−37.259	−1.915	−2.121	−1.709	<0.001	−30.151	−1.486	−1.609	−1.363	<0.001	−43.955	−2.396	−2.698	−2.093	<0.001
155	**Slovenia**	−32.461	−1.609	−1.776	−1.441	<0.001	−29.799	−1.437	−1.749	−1.123	<0.001	−35.433	−1.739	−1.922	−1.556	<0.001
156	**Solomon Islands**	−33.033	−1.705	−1.795	−1.616	<0.001	−26.227	−1.193	−1.304	−1.081	<0.001	−38.303	−2.091	−2.241	−1.940	<0.001
157	**Somalia**	−10.495	−0.820	−0.981	−0.658	<0.001	−4.693	−0.637	−0.809	−0.464	<0.001	−17.587	−1.021	−1.179	−0.862	<0.001
158	**South Africa**	−9.211	−0.577	−1.145	−0.006	0.048	−2.765	−0.357	−0.842	0.130	0.144	−15.556	−0.805	−1.514	−0.091	0.029
159	**South Korea**	3.609	−0.322	−0.648	0.005	0.053	−13.445	−1.326	−1.791	−0.859	<0.001	20.322	0.435	0.145	0.725	0.005
160	**South Sudan**	−11.502	−0.778	−0.916	−0.639	<0.001	−6.562	−0.587	−0.734	−0.439	<0.001	−18.410	−1.030	−1.148	−0.912	<0.001
161	**Spain**	−9.064	−0.240	−0.351	−0.130	<0.001	−9.884	−0.275	−0.377	−0.174	<0.001	−9.827	−0.273	−0.410	−0.136	<0.001
162	**Sri Lanka**	3.644	0.134	0.054	0.213	0.002	1.927	−0.049	−0.141	0.043	0.284	14.376	0.666	0.431	0.902	<0.001
163	**Sudan**	14.619	0.668	0.604	0.733	<0.001	11.517	0.561	0.505	0.617	<0.001	18.767	0.812	0.727	0.896	<0.001
164	**Suriname**	44.523	1.363	1.170	1.556	<0.001	51.903	1.487	1.264	1.712	<0.001	31.581	1.233	1.068	1.399	<0.001
165	**Swaziland**	2.024	0.050	−0.271	0.373	0.751	8.815	0.210	−0.146	0.567	0.236	−5.086	−0.129	−0.428	0.170	0.382
166	**Sweden**	−21.578	−0.570	−0.923	−0.216	0.003	−22.929	−0.605	−0.971	−0.237	0.002	−22.721	−0.663	−1.009	−0.315	0.001
167	**Switzerland**	−7.694	−0.314	−0.372	−0.257	<0.001	5.803	0.132	−0.015	0.279	0.076	−23.675	−1.010	−1.131	−0.889	<0.001
168	**Syria**	−9.702	−0.601	−0.734	−0.468	<0.001	−11.364	−0.710	−0.853	−0.567	<0.001	−5.874	−0.379	−0.505	−0.253	<0.001
169	**Taiwan**	28.380	1.310	0.703	1.920	<0.001	32.427	1.394	0.711	2.082	<0.001	25.861	1.294	0.790	1.801	<0.001
170	**Tajikistan**	20.951	0.268	0.069	0.468	0.010	19.429	0.188	−0.034	0.411	0.094	17.959	0.236	0.051	0.421	0.014
171	**Tanzania**	−10.783	−0.742	−0.966	−0.519	<0.001	−10.671	−0.760	−0.956	−0.563	<0.001	−9.031	−0.631	−0.905	−0.356	<0.001
172	**Thailand**	−23.780	−1.923	−2.601	−1.239	<0.001	−36.433	−2.975	−3.868	−2.073	<0.001	23.666	0.511	0.302	0.720	<0.001
173	**The Bahamas**	−5.994	0.063	−0.207	0.333	0.636	0.425	0.191	0.099	0.284	<0.001	−10.400	−0.060	−0.469	0.351	0.767
174	**The Gambia**	−22.084	−1.192	−1.351	−1.033	<0.001	−22.031	−1.272	−1.475	−1.068	<0.001	−20.764	−1.028	−1.135	−0.921	<0.001
175	**Timor-Leste**	−8.322	−0.318	−0.455	−0.181	<0.001	−2.333	−0.027	−0.182	0.128	0.723	−16.726	−0.746	−0.887	−0.604	<0.001
176	**Togo**	−17.189	−0.952	−1.109	−0.794	<0.001	−14.130	−0.967	−1.172	−0.760	<0.001	−15.614	−0.612	−0.777	−0.448	<0.001
177	**Tonga**	−13.978	−0.675	−0.735	−0.615	<0.001	−12.523	−0.559	−0.613	−0.505	<0.001	−13.946	−0.761	−0.852	−0.670	<0.001
178	**Trinidad and Tobago**	73.595	3.217	2.522	3.916	<0.001	89.509	3.420	2.728	4.115	<0.001	49.287	2.817	2.004	3.636	<0.001
179	**Tunisia**	4.453	0.183	0.167	0.199	<0.001	4.510	0.180	0.160	0.199	<0.001	6.291	0.266	0.249	0.283	<0.001
180	**Turkey**	−4.198	−0.117	−0.273	0.039	0.135	13.771	0.784	0.609	0.960	<0.001	−23.465	−1.305	−1.579	−1.029	<0.001
181	**Turkmenistan**	49.829	1.866	1.660	2.073	<0.001	41.404	1.594	1.358	1.830	<0.001	58.536	2.136	1.939	2.334	<0.001
182	**Uganda**	7.245	−0.036	−0.236	0.164	0.711	30.255	0.743	0.511	0.976	<0.001	−13.041	−0.894	−1.085	−0.703	<0.001
183	**Ukraine**	−32.710	−2.339	−2.761	−1.914	<0.001	−19.735	−1.613	−2.016	−1.209	<0.001	−44.467	−3.128	−3.580	−2.673	<0.001
184	**United Arab Emirates**	33.989	1.320	1.228	1.411	<0.001	28.952	1.175	1.098	1.252	<0.001	46.874	1.674	1.512	1.836	<0.001
185	**United Kingdom**	11.018	0.893	0.635	1.152	<0.001	9.065	0.908	0.641	1.177	<0.001	11.624	0.837	0.576	1.098	<0.001
186	**United States**	−3.427	−0.046	−0.247	0.156	0.645	−9.073	−0.507	−0.718	−0.295	<0.001	1.354	0.438	0.196	0.682	0.001
187	**Uruguay**	14.666	0.597	0.477	0.718	<0.001	20.629	0.935	0.766	1.104	<0.001	10.553	0.346	0.167	0.525	0.001
188	**Uzbekistan**	5.954	0.277	0.220	0.333	<0.001	6.659	0.336	0.245	0.426	<0.001	4.335	0.178	0.145	0.211	<0.001
189	**Vanuatu**	−24.385	−1.178	−1.343	−1.013	<0.001	−15.132	−0.592	−0.723	−0.461	<0.001	−32.431	−1.737	−1.948	−1.525	<0.001
190	**Venezuela**	−5.479	−0.839	−1.222	−0.455	<0.001	1.209	−0.587	−0.994	−0.177	0.007	−11.266	−1.097	−1.560	−0.633	<0.001
191	**Vietnam**	−5.790	−0.072	−0.206	0.063	0.281	−7.583	−0.181	−0.298	−0.063	0.004	−6.086	−0.043	−0.203	0.116	0.582
192	**Virgin Islands, U.S.**	58.206	1.935	1.618	2.253	<0.001	130.464	2.954	2.217	3.695	<0.001	17.516	0.875	0.709	1.041	<0.001
193	**Yemen**	11.817	0.498	0.461	0.535	<0.001	10.135	0.452	0.413	0.492	<0.001	14.898	0.598	0.556	0.639	<0.001
194	**Zambia**	−18.643	−1.413	−1.683	−1.142	<0.001	−4.385	−0.808	−1.071	−0.544	<0.001	−33.386	−2.181	−2.691	−1.669	<0.001
195	**Zimbabwe**	24.168	0.786	0.218	1.358	0.009	37.611	1.146	0.412	1.886	0.003	4.828	0.174	0.050	0.298	0.008

Abbreviations: PC: percentage change; APC: annual percentage change. *p* < 0.001 considered significant.

**Table 7:** Percentage change and annual percentage change of disability adjusted life years of urolithiasis stratified by gender and SDI level.

	Both Male and Female					Male					Female				
	PC	APC	95%CI	95%CI	*p*-Value	PC	APC	95%CI	95%CI	*p*-Value	PC	APC	95%CI	95%CI	*p*-Value
**Global**	−35.862	−1.898	−2.117	−1.679	<0.001	−35.043	−1.812	−1.973	−1.650	<0.001	−37.959	−2.078	−2.383	−1.773	<0.001
**Low SDI**	−16.723	−0.720	−0.851	−0.589	<0.001	−14.591	−0.569	−0.677	−0.461	<0.001	−17.297	−0.821	−1.013	−0.629	<0.001
**Low-middle SDI**	−22.509	−1.053	−1.166	−0.940	<0.001	−20.969	−0.898	−0.985	−0.812	<0.001	−22.656	−1.185	−1.370	−0.999	<0.001
**Middle SDI**	−43.070	−2.494	−2.705	−2.283	<0.001	−42.137	−2.376	−2.535	−2.216	<0.001	−44.517	−2.679	−3.004	−2.352	<0.001
**High-middle SDI**	−50.297	−3.096	−3.413	−2.777	<0.001	−50.098	−3.004	−3.254	−2.753	<0.001	−52.281	−3.343	−3.748	−2.936	<0.001
**High SDI**	−15.837	−0.480	−0.694	−0.266	<0.001	−17.428	−0.628	−0.821	−0.435	<0.001	−16.001	−0.381	−0.635	−0.127	0.005

Abbreviations: PC: percentage change; APC: annual percentage change; SDI: sociodemographic index. *p* < 0.001 considered significant.

## Data Availability

The datasets generated during and/or analyzed during the current study are available from the Global Health Data Exchange query tool (http://ghdx.healthdata.org/gbd-results-tool (accessed on 1 December 2021)).

## Supplementary Materials

The following supporting information can be downloaded at: https://www.mdpi.com/article/10.3390/jcm12031048/s1, Figure S1: Flowchart, urolithiasis incidence to DALY estimation; Table S1: GATHER checklist; Table S2: The geographical coverage of urolithiasis data by measure in GBD 2019; Table S3: Covariates selected for CODEm for urolithiasis and expected direction of covariate; Table S4: Total number of site years by cause and source type for GBD 2019; Table S5: Results for CODEm model testing; Table S6: Comparison of GBD 2016 and GBD 2019 covariates used and level of covariates; Table S7: Socio-Demographic Index groupings by location, based on 2019 values. References [42,43,44,45,46] are cited in the Supplementary Materials.
