# Supplementary material for: Global, Regional, and National Incidence and Disability-Adjusted Life-Years for Urolithiasis in 195 Countries and Territories, 1990–2019: Results from the Global Burden of Disease Study 2019
Source: J Clin Med. 2023 Jan 29;12(3):1048. doi: 10.3390/jcm12031048 (PMC9918205; doi:10.3390/jcm12031048)

Supplementary materials

GBD estimates of burden are updated each iteration based on updated data sources and methodologies. Global data sources used in fatal and nonfatal estimation of urolithiasis are available from the GBD Input Sources Tool [1].

Case definition

Formation of stones in any part of the urinary tract, usually in the kidney, urinary bladder, or the ureter. For urolithiasis, the ICD 10 codes are N20-N23.0, and ICD 9 codes are 592-592.9, 594-594.9, 788.0.

Input data

For GBD 2010, a systematic review of the prevalence of AU throughout the world was conducted. This search was updated for GBD 2013 and again for GBD 2016. A PubMed search was conducted using the following search terms: (Urolithiasis [Title/Abstract] OR Kidney Stones [Title/Abstract]) AND (Prevalence [Title/Abstract] OR Incidence [Title/Abstract]) AND ("2013/01/01" [PDAT]: "3000" [PDAT]) AND "humans" [MeSH Terms].

Four new studies were added based on this systematic review. The Table S2 below shows the geographical coverage of urolithiasis data by measure in GBD 2019. Claims data for the USA, Philippines, Taiwan, and New Zealand were included. Hospital inpatient data were also included. Inpatient data points with an age-standardised incidence rate greater than two median absolute deviations from the median of the age-standardised incidence rate for all inpatient data were marked as outliers and excluded from analysis.

Modelling strategy

The estimation strategy used for urolithiasis is largely similar to methods used in GBD 2016. A standard CODEm model including location-level covariates was used to model deaths due to urolithiasis. Age-restrictions for death estimations secondary to urolithiasis include 5 years for lower bound, 95+ for upper bound. Iterations of models were assessed at the location/year/age group/sex level to determine whether data points merited exclusion via outliering. Unadjusted death estimates were adjusted using CoDCorrect to produce final estimates of YLLs. The estimates are limited by a paucity of data for regions such as Eastern and Central sub-Saharan Africa.

Covariates are selected independently for each sub-model and the selection is based on an algorithm that captures biologically plausible relationships between the covariates and LRI mortality and provides a diversity of possible models. Each model includes all combinations of covariates if the direction of effect is along the assumed direction and the coefficient is significant at the $p < 0.05$ level. Also, if adding a higher level covariate changes the significance of a level one to non-significant or an implausible direction, it will be dropped from the set. (Table S3)

CoD data types

The CoD database contains seven types of data sources (Table S4): vital registration (VR), verbal autopsy (VA), cancer registry (CR), police records, sibling history, surveillance, and survey/census. The highest-quality data have detailed demographic group characteristics and detailed CoD across the time series. Data from countries with complete VR systems are considered to be high-quality. For countries with incomplete VR systems, vital statistics for causes of death may be supplemented with other data types to provide cause-specific estimates.

Decomposition analysis

We first used the decomposition methodology of Das Gupta [2–4] to decompose urolithiasis DALYs by population age structure, population growth, and epidemiologic changes. The number of DALYs at each location was obtained from the following formula:

$$\mathrm{DALY}_{ay,\,py,\,ey} = \sum_{i=1}^{20}(a_{i,\,y} * p_{y} * e_{i,\,y})$$

Where $\mathrm{DALY}_{ay,\,py,\,ey}$ represented DALYs based on the factors of age structure, population, and DALYs rate for specific year y; $a_{i\,y}$ represents the proportion of population for the age category I (amongst the 20 age categories) in given year y; p_{y} represents the total population in given year y; and $e_{i,\,y}$ represents DALYs rate given age category i in year y. The contribution of each factor to the change in DALYs from 1990 to 2019 was defined by the effect of one factor changing while the other factors were held constant. For example, the effect of age structure was calculated as:

$$[(\mathrm{DALY}_{a2019,\,p1990,\,e1990} + \mathrm{DALY}_{a2019,\,p2019,\,e2019})/3 + (\mathrm{DALY}_{a2019,\,p1990,\,e2019} + \mathrm{DALY}_{a2019,\,p2019,\,e1990})/6] -$$
$$[(\mathrm{DALY}_{a1990,\,p2019,\,e2019} + \mathrm{DALY}_{a1990,\,p1990,\,e1990})/3 + (\mathrm{DALY}_{a1990,\,p2019b,\,e1990} + \mathrm{DALY}_{a1990,\,p1990,\,e2019})/6]$$

Health Care Access and Quality

The Health Care Access and Quality (HAQ) index was developed by the GBD study group[4]; it is based on risk, age and population-standardized cause-specific death rates of 32 GBD causes of morality amenable to personal healthcare. It provides a summary measure on a scale of 0 to 100 to facilitate comparison of personal health-care access and quality by geography and over time [4]

Because SDI may confound the association between age-standardized DALYs rates and HAQ, we used all data available for DALYs rates and HAQ in 1990, 1995, 2000, 2005, 2010, and 2015 to build a generalized estimating equation with identity link and an independence working correlation structure. To account for possible non-linear relationship between HAQ/SDI and DALYs, HAQ was treat as restricted cubic spline function while controlling for SDI (which was also treated as a spline function). Knots were placed every quartile for the cubic function. In addition, because the association of HAQ and DALYs may vary by GBD region, we examined the relationship within GBD region by placing it as the indicator variable in the models [5].

References

1. *Global Burden of Disease Study 2019 (GBD 2019) Data Input Sources Tool*; Institute for Health Metrics and Evaluation: Seattle, WA, USA, 2019. Available online: https://ghdx.healthdata.org/gbd-2019/data-input-sources (accessed on 1 December 2022).
2. Das Gupta, P. *Standardization and Decomposition of Rates: A User's Manual*; U.S. Department of Commerce, Economics and Statistics Administration, Bureau of the Census: Washington, DC, USA, 1993; pp. 19–36.
3. Das Gupta, P. Standardization and Decomposition of Rates from Cross-Classified Data. *Genus* **1994**, *50*, 171–196.
4. Chevan, A.; Sutherland, M. Revisiting Das Gupta: refinement and extension of standardization and decomposition. *Demography* **2009**, 46, 429–449.
5. Bowe, B.; Xie, Y.; Li, T.; Yan, Y.; Xian, H.; Al-Aly, Z. Particulate Matter Air Pollution and the Risk of Incident CKD and Progression to ESRD. *J. Am. Soc. Nephrol.* **2018**, *29*, 218–230.

Table S1: GATHER checklist

	Checklist item	Reported on page #
Objectives and funding		
1	Define the indicator(s), populations (including age, sex, and geographic entities), and time period(s) for which estimates were made.	Main text (Methods) and Supplement
2	List the funding sources for the work.	Funding sources listed in paper
Data Inputs		
For all data inputs from multiple sources that are synthesized as part of the study:		
3	Describe how the data were identified and how the data were accessed.	Main text (Methods) and Supplement
4	Specify the inclusion and exclusion criteria. Identify all ad-hoc exclusions.	Main text (Methods) and Supplement
5	Provide information on all included data sources and their main characteristics. For each data source used, report reference information or contact name/institution, population represented, data collection method, year(s) of data collection, sex and age range, diagnostic criteria or measurement method, and sample size, as relevant.	http://ghdx.healthdata.org/gbd-2019/data-input-sources
6	Identify and describe any categories of input data that have potentially important biases (e.g., based on characteristics listed in item 5).	Supplement
For data inputs that contribute to the analysis but were not synthesized as part of the study:		
7	Describe and give sources for any other data inputs.	http://ghdx.healthdata.org/gbd-2019/data-input-sources
For all data inputs:		
8	Provide all data inputs in a file format from which data can be efficiently extracted (e.g., a spreadsheet rather than a PDF), including all relevant meta-data listed in item 5. For any data inputs that cannot be shared because of ethical or legal reasons, such as third-party ownership, provide a contact name or the name of the institution that retains the right to the data.	http://ghdx.healthdata.org/gbd-2019/data-input-sources
Data analysis		
9	Provide a conceptual overview of the data analysis method. A diagram may be helpful.	Figure S1: Flowchart, urolithiasis mortality estimation<br>Figure S2. Flowchart, urolithiasis incidence to DALY estimation
10	Provide a detailed description of all steps of the analysis, including mathematical formulae. This description should cover, as relevant, data cleaning, data pre-processing, data adjustments and weighting of data sources, and mathematical or statistical model(s).	Main text (Methods) and Supplement

11	Describe how candidate models were evaluated and how the final model(s) were selected.	Table S3: Covariates selected for CODEm for urolithiasis and expected direction of covariate
12	Provide the results of an evaluation of model performance, if done, as well as the results of any relevant sensitivity analysis.	Table S5 : Results for CODEm model testing
13	Describe methods for calculating uncertainty of the estimates. State which sources of uncertainty were, and were not, accounted for in the uncertainty analysis.	Supplement
14	State how analytic or statistical source code used to generate estimates can be accessed.	http://ghdx.healthdata.org/gbd-2019/code
Results and Discussion		
15	Provide published estimates in a file format from which data can be efficiently extracted.	GBD 2019 estimates are available online (https://vizhub.healthdata.org/gbd-compare/).
16	Report a quantitative measure of the uncertainty of the estimates (e.g., uncertainty intervals).	Done
17	Interpret results in light of existing evidence. If updating a previous set of estimates, describe the reasons for changes in estimates.	Table S6: Comparison of GBD 2016 and GBD 2019 covariates used and level of covariates
18	Discuss limitations of the estimates. Include a discussion of any modelling assumptions or data limitations that affect interpretation of the estimates.	Main text (Limitations)

Table S2. The geographical coverage of urolithiasis data by measure in GBD 2019

	Incidence	Prevalence
Site-years (total)	1420	5
Number of countries with data	45	4
Number of GBD regions with data (out of 21 regions)	16	4
Number of GBD super-regions with data (out of 7 super-regions)	7	3

Table S3: Covariates selected for CODEm for urolithiasis and expected direction of covariate

Level	Covariate	Direction
1	Temperature (90 th percentile)	+
2	Animal fat (kcal per capita)	+
	Fruits (kcal per capita)	−
	Vegetables (kcal per capita)	−
	Red meat (kcal per capita)	+
	Healthcare access and quality index	−
3	Socio-demographic Index	0
	Log LDI ($I per capita)	−

Table S4. Total number of site years by cause and source type for GBD 2019

Cause	Level	Vital Registration	Vital Registration-Sample	Verbal Autopsy	Surveillance	Survey/Census	Sibling History	Cancer Registry	Police Records
Urolithiasis	4	17639	793	170					

Table S5: Results for CODEm model testing

Cause	Sex	Age Start	Age End	RMSE In	RMSE out	Trend In	Trend Out	Coverage In	Coverage Out
Urolithiasis [Global]	Female	5-9 years	95+ years	0.381017	0.718165	0.248015	0.242039	0.995382	0.977628
Urolithiasis [Data Rich]	Female	5-9 years	95+ years	0.262905	0.412775	0.194587	0.213463	0.998555	0.997319
Urolithiasis [Global]	Male	5-9 years	95+ years	0.459024	0.817927	0.288235	0.290452	0.991246	0.973955
Urolithiasis [Data Rich]	Male	5-9 years	95+ years	0.294543	0.446144	0.22309	0.221486	0.996739	0.994922

Table S6: Comparison of GBD 2016 and GBD 2019 covariates used and level of covariates

Cause	Sex	Covariate	Level 1 2019	Level 2 2019	Level 3 2019	Level 1 2016	Level 2 2016	Level 3 2016
**Urolithiasis**	Female	LDI (I$ per capita)			X			X
**Urolithiasis**	Male	Healthcare access and quality index	X			X		
**Urolithiasis**	Female	Socio-demographic Index			X			X
**Urolithiasis**	Male	Socio-demographic Index			X			X
**Urolithiasis**	Female	red meats adjusted(g)		X			X	
**Urolithiasis**	Male	red meats adjusted(g)		X			X	
**Urolithiasis**	Male	red meats adjusted(g)		X		X		
**Urolithiasis**	Female	vegetables adjusted(g)		X			X	
**Urolithiasis**	Male	red meats adjusted(g)	X			X		
**Urolithiasis**	Female	fruits adjusted(g)		X			X	
**Urolithiasis**	Male	fruits adjusted(g)		X			X	
**Urolithiasis**	Male	Healthcare access and quality index	X				X	

**Urolithiasis**	Female	90th percentile climatic temperature in the given country-year.	X			X		
**Urolithiasis**	Male	90th percentile climatic temperature in the given country-year.	X			X		
**Urolithiasis**	Male	red meats adjusted(g)	X				X	
**Urolithiasis**	Male	Healthcare access and quality index		X		X		
**Urolithiasis**	Male	vegetables adjusted(g)		X			X	
**Urolithiasis**	Female	Healthcare access and quality index	X			X		
**Urolithiasis**	Male	LDI (I$ per capita)			X			X
**Urolithiasis**	Male	Education (years per capita)			X			X
**Urolithiasis**	Female	Education (years per capita)			X			X
**Urolithiasis**	Male	Healthcare access and quality index		X				

Table S7. Socio-Demographic Index groupings by location, based on 2019 values

Location Name	2019 SDI Index Value	SDI Quintile
Global	0.652205351	
Central Europe, Eastern Europe, and Central Asia	0.765735064	
Central Asia	0.672778523	
Armenia	0.702021479	High-middle SDI
Azerbaijan	0.701169598	High-middle SDI
Georgia	0.699719344	High-middle SDI
Kazakhstan	0.735474229	High-middle SDI
Kyrgyzstan	0.606646902	Low-middle SDI
Mongolia	0.661854015	Middle SDI
Tajikistan	0.522612209	Low-middle SDI
Turkmenistan	0.696418617	Middle SDI
Uzbekistan	0.629546531	Middle SDI
Central Europe	0.813976167	
Albania	0.684614242	Middle SDI
Bosnia and Herzegovina	0.712609905	High-middle SDI
Bulgaria	0.79173721	High-middle SDI
Croatia	0.824844721	High SDI
Czech Republic	0.850980459	High SDI
Hungary	0.816804322	High-middle SDI
Macedonia	0.75436361	High-middle SDI
Montenegro	0.788188778	High-middle SDI
Poland	0.84377326	High SDI
Romania	0.784193905	High-middle SDI
Serbia	0.75179332	High-middle SDI
Slovakia	0.841690487	High SDI
Slovenia	0.860279598	High SDI
Eastern Europe	0.785420363	
Belarus	0.772665439	High-middle SDI
Estonia	0.857709406	High SDI
Latvia	0.825131484	High SDI
Lithuania	0.840877452	High SDI
Moldova	0.675572758	Middle SDI
Russian Federation	0.791738063	High-middle SDI
Ukraine	0.740061596	High-middle SDI
High-income	0.854428248	
Australasia	0.868509969	
Australia	0.873188291	High SDI

Location Name	2019 SDI Index Value	SDI Quintile
New Zealand	0.842273544	High SDI
High-income Asia Pacific	0.86894981	
Brunei	0.856240565	High SDI
Japan	0.865093512	High SDI
Aichi	0.874998978	High SDI
Akita	0.829009097	High SDI
Aomori	0.825175188	High SDI
Chiba	0.859238574	High SDI
Ehime	0.838399264	High SDI
Fukui	0.852281964	High SDI
Fukuoka	0.855307883	High SDI
Fukushima	0.830930555	High SDI
Gifu	0.84923591	High SDI
Gunma	0.850963336	High SDI
Hiroshima	0.862595627	High SDI
Hokkaido	0.841522308	High SDI
Hyogo	0.859765235	High SDI
Ibaraki	0.850665189	High SDI
Ishikawa	0.856039392	High SDI
Iwate	0.825241842	High SDI
Kagawa	0.849935485	High SDI
Kagoshima	0.829680279	High SDI
Kanagawa	0.874939342	High SDI
Kochi	0.825446834	High SDI
Kumamoto	0.831536501	High SDI
Kyoto	0.87256007	High SDI
Mie	0.853567757	High SDI
Miyagi	0.850313137	High SDI
Miyazaki	0.823112655	High SDI
Nagano	0.851209245	High SDI
Nagasaki	0.826141869	High SDI
Nara	0.847998888	High SDI
Niigata	0.843300137	High SDI
Oita	0.845989117	High SDI
Okayama	0.855866898	High SDI
Okinawa	0.817915416	High SDI
Osaka	0.872366437	High SDI
Saga	0.833665065	High SDI
Saitama	0.8520121	High SDI

Shiga	0.870844353	High SDI
**Location Name**	**2019 SDI Index Value**	**SDI Quintile**
Shimane	0.831040466	High SDI
Shizuoka	0.858790953	High SDI
Tochigi	0.853264467	High SDI
Tokushima	0.845285	High SDI
Tokyo	0.924328028	High SDI
Tottori	0.83436659	High SDI
Toyama	0.859824207	High SDI
Wakayama	0.839775092	High SDI
Yamagata	0.831923683	High SDI
Yamaguchi	0.849441807	High SDI
Yamanashi	0.854296098	High SDI
South Korea	0.871955704	High SDI
Singapore	0.872215248	High SDI
High-income North America	0.868169406	
Canada	0.882086227	High SDI
Greenland	0.760075292	High-middle SDI
United States	0.86662166	High SDI
Alabama	0.837233514	High SDI
Alaska	0.86060992	High SDI
Arizona	0.845107314	High SDI
Arkansas	0.826148933	High SDI
California	0.872398094	High SDI
Colorado	0.882128544	High SDI
Connecticut	0.906486727	High SDI
Delaware	0.873744053	High SDI
District of Columbia	0.890203139	High SDI
Florida	0.863631092	High SDI
Georgia	0.848426298	High SDI
Hawaii	0.872290363	High SDI
Idaho	0.840713155	High SDI
Illinois	0.879386003	High SDI
Indiana	0.84792909	High SDI
Iowa	0.8704793	High SDI
Kansas	0.864464964	High SDI
Kentucky	0.83130395	High SDI
Louisiana	0.834894869	High SDI
Maine	0.872309993	High SDI
Maryland	0.895667105	High SDI

Massachusetts	0.913307727	High SDI
Michigan	0.867717003	High SDI
**Location Name**	**2019 SDI Index Value**	**SDI Quintile**
Minnesota	0.892987345	High SDI
Mississippi	0.818942009	High SDI
Missouri	0.85325798	High SDI
Montana	0.863383139	High SDI
Nebraska	0.87308561	High SDI
Nevada	0.847315003	High SDI
New Hampshire	0.904304115	High SDI
New Jersey	0.899124902	High SDI
New Mexico	0.835274776	High SDI
New York	0.893442339	High SDI
North Carolina	0.84978326	High SDI
North Dakota	0.879820384	High SDI
Ohio	0.858271211	High SDI
Oklahoma	0.838181089	High SDI
Oregon	0.870700326	High SDI
Pennsylvania	0.878553277	High SDI
Rhode Island	0.890036984	High SDI
South Carolina	0.846024965	High SDI
South Dakota	0.860188872	High SDI
Tennessee	0.836985155	High SDI
Texas	0.837777472	High SDI
Utah	0.855766922	High SDI
Vermont	0.89559193	High SDI
Virginia	0.885122306	High SDI
Washington	0.88440099	High SDI
West Virginia	0.824706332	High SDI
Wisconsin	0.87773172	High SDI
Wyoming	0.869345173	High SDI
Southern Latin America	0.720171023	
Argentina	0.710150584	High-middle SDI
Chile	0.748081344	High-middle SDI
Uruguay	0.706753401	High-middle SDI
Western Europe	0.856820142	
Andorra	0.901838419	High SDI
Austria	0.866029424	High SDI
Belgium	0.886479194	High SDI
Cyprus	0.86457342	High SDI

Denmark	0.917864091	High SDI
Finland	0.892872363	High SDI
France	0.864667258	High SDI
**Location Name**	**2019 SDI Index Value**	**SDI Quintile**
Germany	0.869902009	High SDI
Greece	0.816993531	High SDI
Iceland	0.907023083	High SDI
Ireland	0.882181159	High SDI
Israel	0.81594436	High-middle SDI
Italy	0.843401161	High SDI
Luxembourg	0.915748227	High SDI
Malta	0.835898842	High SDI
Netherlands	0.911855053	High SDI
Norway	0.910905362	High SDI
Portugal	0.777927627	High-middle SDI
Spain	0.824616837	High SDI
Sweden	0.883490275	High SDI
Stockholm	0.914447593	High SDI
Sweden except Stockholm	0.872833379	High SDI
Switzerland	0.888752501	High SDI
United Kingdom	0.843093074	High SDI
England	0.848869853	High SDI
East Midlands	0.83007704	High SDI
East of England	0.840300066	High SDI
Greater London	0.894369062	High SDI
North East England	0.820735615	High SDI
North West England	0.833664296	High SDI
South East England	0.856169812	High SDI
South West England	0.841270041	High SDI
West Midlands	0.829368047	High SDI
Yorkshire and the Humber	0.829690925	High SDI
Northern Ireland	0.835352065	High SDI
Scotland	0.805372811	High SDI
Wales	0.805748561	High SDI
Latin America and Caribbean	0.639865451	
Andean Latin America	0.628313955	
Bolivia	0.587409304	Low-middle SDI
Ecuador	0.635566909	Middle SDI
Peru	0.635787809	Middle SDI
Caribbean	0.637604561	

Antigua and Barbuda	0.715130979	High-middle SDI
The Bahamas	0.75556215	High-middle SDI
Barbados	0.739423177	High-middle SDI
Belize	0.602243591	Low-middle SDI
**Location Name**	**2019 SDI Index Value**	**SDI Quintile**
Bermuda	0.80545317	High-middle SDI
Cuba	0.687667664	Middle SDI
Dominica	0.68658657	Middle SDI
Dominican Republic	0.592640504	Low-middle SDI
Grenada	0.640418422	Middle SDI
Guyana	0.583747015	Low-middle SDI
Haiti	0.441665969	Low SDI
Jamaica	0.678532504	Middle SDI
Puerto Rico	0.812984477	High-middle SDI
Saint Lucia	0.652614198	Middle SDI
Saint Vincent and the Grenadines	0.608304473	Middle SDI
Suriname	0.64099299	Middle SDI
Trinidad and Tobago	0.698405348	Middle SDI
Virgin Islands, U.S.	0.806568682	High-middle SDI
Central Latin America	0.623192305	
Colombia	0.633692252	Middle SDI
Costa Rica	0.662129526	Middle SDI
El Salvador	0.59309467	Low-middle SDI
Guatemala	0.524214498	Low-middle SDI
Honduras	0.512339813	Low-middle SDI
Mexico	0.628360997	Middle SDI
Aguascalientes	0.659089353	Middle SDI
Baja California	0.656785464	Middle SDI
Baja California Sur	0.658976353	Middle SDI
Campeche	0.615914899	Middle SDI
Chiapas	0.53276266	Middle SDI
Chihuahua	0.638589391	Middle SDI
Coahuila	0.645326148	Middle SDI
Colima	0.65420353	Middle SDI
Durango	0.623979236	Middle SDI
Guanajuato	0.62129178	Middle SDI
Guerrero	0.562442968	Middle SDI
Hidalgo	0.587458446	Middle SDI
Jalisco	0.648991934	Middle SDI

Mexico	0.635428465	Middle SDI
Mexico City	0.715772109	Middle SDI
Michoacan de Ocampo	0.58646838	Middle SDI
Morelos	0.635471941	Middle SDI
Nayarit	0.620025881	Middle SDI
**Location Name**	**2019 SDI Index Value**	**SDI Quintile**
Nuevo Leon	0.677420872	Middle SDI
Oaxaca	0.560543467	Middle SDI
Puebla	0.584252823	Middle SDI
Queretaro	0.639127345	Middle SDI
Quintana Roo	0.626303085	Middle SDI
San Luis Potosi	0.620944629	Middle SDI
Sinaloa	0.648534168	Middle SDI
Sonora	0.650495685	Middle SDI
Tabasco	0.611463527	Middle SDI
Tamaulipas	0.647006129	Middle SDI
Tlaxcala	0.604441163	Middle SDI
Veracruz de Ignacio de la Llave	0.591994	Middle SDI
Yucatan	0.63033024	Middle SDI
Zacatecas	0.607654208	Middle SDI
Nicaragua	0.529616174	Low-middle SDI
Panama	0.677043867	Middle SDI
Venezuela	0.655413104	Middle SDI
Tropical Latin America	0.662126282	
Brazil	0.663312473	Middle SDI
Acre	0.601605235	Low-middle SDI
Alagoas	0.555715012	Low-middle SDI
Amapa	0.658517629	Middle SDI
Amazonas	0.629315711	Middle SDI
Bahia	0.591019766	Low-middle SDI
Ceara	0.599501511	Low-middle SDI
Distrito Federal	0.79189036	High-middle SDI
Espirito Santo	0.676646695	Middle SDI
Goias	0.650146424	Middle SDI
Maranhao	0.507040138	Low-middle SDI
Mato Grosso	0.662454796	Middle SDI
Mato Grosso do Sul	0.650210546	Middle SDI
Minas Gerais	0.660795264	Middle SDI
Para	0.578664243	Low-middle SDI

Paraiba	0.574462555	Low-middle SDI
Parana	0.682436727	Middle SDI
Pernambuco	0.593552542	Low-middle SDI
Piaui	0.551619925	Low-middle SDI
Rio de Janeiro	0.708855843	High-middle SDI
Rio Grande do Norte	0.605294307	Low-middle SDI
**Location Name**	**2019 SDI Index Value**	**SDI Quintile**
Rio Grande do Sul	0.6927427	Middle SDI
Rondonia	0.621702361	Middle SDI
Roraima	0.646354751	Middle SDI
Santa Catarina	0.702495682	High-middle SDI
Sao Paulo	0.7200519	High-middle SDI
Sergipe	0.615627706	Middle SDI
Tocantins	0.610879077	Middle SDI
Paraguay	0.618769591	Middle SDI
North Africa and Middle East	0.638603537	
North Africa and Middle East	0.638603537	
Afghanistan	0.290254968	Low SDI
Algeria	0.695849021	Middle SDI
Bahrain	0.712258604	High-middle SDI
Egypt	0.604307711	Low-middle SDI
Iran	0.700086759	High-middle SDI
Iraq	0.584823813	Low-middle SDI
Jordan	0.696845045	Middle SDI
Kuwait	0.785593198	High-middle SDI
Lebanon	0.729621127	High-middle SDI
Libya	0.760934217	High-middle SDI
Morocco	0.579231309	Low-middle SDI
Palestine	0.541353069	Low-middle SDI
Oman	0.743531097	High-middle SDI
Qatar	0.765715882	High-middle SDI
Saudi Arabia	0.7790137	High-middle SDI
Sudan	0.477915229	Low-middle SDI
Syria	0.611084286	Middle SDI
Tunisia	0.675428611	Middle SDI
Turkey	0.729481001	High-middle SDI
United Arab Emirates	0.794722025	High-middle SDI
Yemen	0.429504407	Low SDI
South Asia	0.533975763	

South Asia	0.533975763	
Bangladesh	0.457988721	Low SDI
Bhutan	0.569907913	Low-middle SDI
India	0.550242018	Low-middle SDI
Nepal	0.428511471	Low SDI
Pakistan	0.492158484	Low-middle SDI
Southeast Asia, East Asia, and Oceania	0.685403755	
**Location Name**	**2019 SDI Index Value**	**SDI Quintile**
East Asia	0.708630758	
China	0.707319288	High-middle SDI
North Korea	0.537679957	Low-middle SDI
Taiwan	0.86418562	High SDI
Oceania	0.470985744	
American Samoa	0.701859796	High-middle SDI
Federated States of Micronesia	0.575251612	Low-middle SDI
Fiji	0.641435501	Middle SDI
Guam	0.794193119	High-middle SDI
Kiribati	0.426768011	Low SDI
Marshall Islands	0.550457832	Low-middle SDI
Northern Mariana Islands	0.75781722	High-middle SDI
Papua New Guinea	0.418998443	Low SDI
Samoa	0.576375166	Low-middle SDI
Solomon Islands	0.425018528	Low SDI
Tonga	0.624951156	Middle SDI
Vanuatu	0.475309121	Low-middle SDI
Southeast Asia	0.640717246	
Cambodia	0.481619391	Low-middle SDI
Indonesia	0.647611359	Middle SDI
Aceh	0.640414411	Middle SDI
Bali	0.646777358	Middle SDI
Bangka-Belitung Islands	0.637063919	Middle SDI
Banten	0.636136405	Middle SDI
Bengkulu	0.605588458	Low-middle SDI
Gorontalo	0.556881893	Low-middle SDI
Jakarta	0.795041917	High-middle SDI
Jambi	0.640546524	Middle SDI
West Java	0.635672599	Middle SDI
Central Java	0.606724047	Middle SDI

East Java	0.64169154	Middle SDI
West Kalimantan	0.589201584	Low-middle SDI
South Kalimantan	0.623798672	Middle SDI
Central Kalimantan	0.641894718	Middle SDI
East Kalimantan	0.746595227	High-middle SDI
North Kalimantan	0.755952734	High-middle SDI
Riau Islands	0.727599596	High-middle SDI
Lampung	0.616299987	Middle SDI
Maluku	0.555610326	Low-middle SDI
**Location Name**	**2019 SDI Index Value**	**SDI Quintile**
North Maluku	0.546157963	Low-middle SDI
West Nusa Tenggara	0.556566054	Low-middle SDI
East Nusa Tenggara	0.518912804	Low-middle SDI
Papua	0.587862719	Low-middle SDI
West Papua	0.683007739	Middle SDI
Riau	0.713955299	High-middle SDI
West Sulawesi	0.559336878	Low-middle SDI
South Sulawesi	0.610967812	Middle SDI
Central Sulawesi	0.612199879	Middle SDI
Southeast Sulawesi	0.596388581	Low-middle SDI
North Sulawesi	0.651649236	Middle SDI
West Sumatra	0.640858055	Middle SDI
South Sumatra	0.642344679	Middle SDI
North Sumatra	0.653390877	Middle SDI
Yogyakarta	0.65012062	Middle SDI
Laos	0.518788871	Low-middle SDI
Malaysia	0.759248836	High-middle SDI
Maldives	0.655286841	Middle SDI
Mauritius	0.720190502	High-middle SDI
Myanmar	0.555817824	Low-middle SDI
Philippines	0.617174396	Middle SDI
Sri Lanka	0.679706328	Middle SDI
Seychelles	0.692334035	Middle SDI
Thailand	0.684276785	Middle SDI
Timor-Leste	0.504842989	Low-middle SDI
Vietnam	0.606829222	Middle SDI
Sub-Saharan Africa	0.445980066	
Central Sub-Saharan Africa	0.45690943	
Angola	0.460535938	Low-middle SDI
Central African Republic	0.334449009	Low SDI

Congo	0.574129526	Low-middle SDI
Democratic Republic of the Congo	0.364453165	Low SDI
Equatorial Guinea	0.62522322	Middle SDI
Gabon	0.650559028	Middle SDI
Eastern Sub-Saharan Africa	0.387060963	
Burundi	0.309705632	Low SDI
Comoros	0.434289553	Low SDI
Djibouti	0.484750347	Low-middle SDI
Eritrea	0.408790995	Low SDI
**Location Name**	**2019 SDI Index Value**	**SDI Quintile**
Ethiopia	0.334181415	Low SDI
Kenya	0.499471993	Low-middle SDI
Madagascar	0.330760552	Low SDI
Malawi	0.349345085	Low SDI
Mozambique	0.340470577	Low SDI
Rwanda	0.40744149	Low SDI
Somalia	0.234806633	Low SDI
South Sudan	0.274705978	Low SDI
Tanzania	0.412207128	Low SDI
Uganda	0.387738241	Low SDI
Zambia	0.472213354	Low-middle SDI
Southern Sub-Saharan Africa	0.639979771	
Botswana	0.663238118	Middle SDI
Lesotho	0.493356884	Low-middle SDI
Namibia	0.615792035	Middle SDI
South Africa	0.676542582	Middle SDI
Swaziland	0.577699713	Low-middle SDI
Zimbabwe	0.463195841	Low-middle SDI
Western Sub-Saharan Africa	0.441032713	
Benin	0.373374857	Low SDI
Burkina Faso	0.283938202	Low SDI
Cameroon	0.482039386	Low-middle SDI
Cape Verde	0.549086441	Low-middle SDI
Chad	0.252901641	Low SDI
Cote d’Ivoire	0.412139874	Low SDI
The Gambia	0.404759628	Low SDI
Ghana	0.536972566	Low-middle SDI
Guinea	0.324710505	Low SDI
Guinea-Bissau	0.348986787	Low SDI

Liberia	0.328416338	Low SDI
Mali	0.266900909	Low SDI
Mauritania	0.470565798	Low-middle SDI
Niger	0.190617687	Low SDI
Nigeria	0.49339389	Low-middle SDI
Sao Tome and Principe	0.488258275	Low-middle SDI
Senegal	0.373026564	Low SDI
Sierra Leone	0.357159036	Low SDI
Togo	0.413313302	Low SDI

Figure S1. Flowchart, urolithiasis incidence to DALY estimation

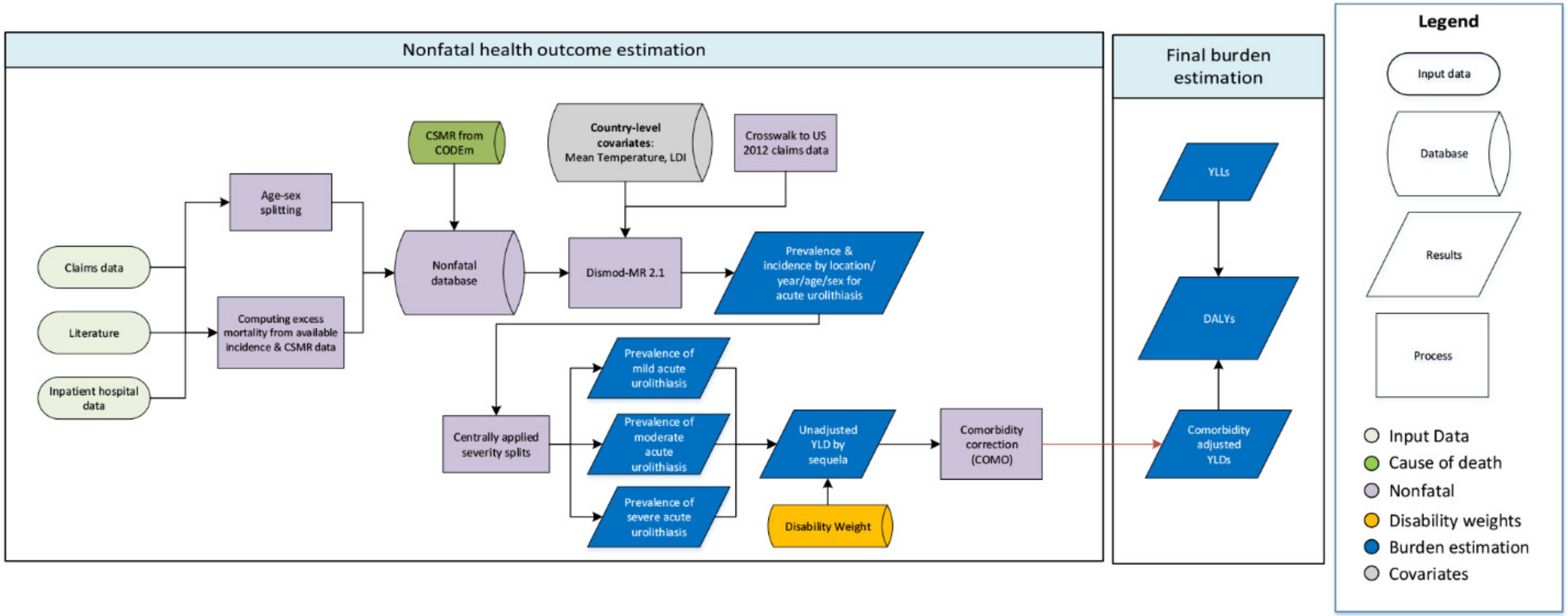

Supplement: Supplementary file 1 [file jcm-12-01048-s001.zip › jcm-2124578-supplementary.pdf]
